# B-Bridge Regulated Asymmetric Dual-Atomic Catalysts for Synergistically Enhanced Styrene Mineralization and CO_2_ Reduction

**DOI:** 10.1007/s40820-025-01820-2

**Published:** 2025-06-23

**Authors:** Xiai Zhang, Zhongshuang Xu, Xinwei Zhang, Jingquan Wang, Dan Liu, Huanran Miao, Tong Wang, Zhimao Yang, Qikui Fan, Chuncai Kong

**Affiliations:** MOE Key Laboratory for Non-Equilibrium Synthesis and Modulation of Condensed Matter, Key Laboratory for Advanced Materials and Mesoscopic Physics of Shaanxi Province, School of Physics, Xi’an Jiaotong University, Xi’an, 710049 People’s Republic of China

**Keywords:** B electron bridge, Dual-atom sites, Microenvironment, Carbon cycle utilization, CO_2_ reduction

## Abstract

Developing innovative resource utilization strategies to achieve sustainable recycling of waste-to-fuel is highly desirable, yet the design of cost-effective bifunctional catalysts with dual high-efficiency remains unexplored. While the Fenton-like reaction relies on enhancing peroxymonosulfate (PMS) adsorption and accelerating interfacial electron transfer to improve kinetic rates, CO_2_ reduction is constrained by sluggish kinetics and competing hydrogen evolution reaction. Herein, we construct a bifunctional catalyst (NiFe-BNC) featuring dual-atomic active sites by introducing boron atoms into a biomass-derived chitosan substrate rich in functional groups, which optimizes atomic coordination environments. In situ experiments and density functional theory calculations reveal that B-atom modulation facilitates carbon substrate defect enrichment, while the charge-tuning effect between metal sites and "boron electron bridge" optimizes PMS adsorption configurations. This synergistic effect facilitates the interfacial electron transfer and enhances the CO_2_ adsorption capacity of NiFe-BNC by 6 times that of NiFe-NC. The obtained NiFe-BNC exhibits significantly enhanced catalytic activity and selectivity, realizing 99% efficient degradation of volatile organic pollutants in the flowing phase within 2 h and stable mineralization exceeding 60%, while achieving a large current density of 1000 mA cm^−2^ and CO Faraday efficiency of 98% in the flow electrolytic cell. This work innovatively paves a new way for the rational design of cost-effective functional catalysts to achieve carbon cycle utilization.

**Supplementary Information:**

The online version contains supplementary material available at 10.1007/s40820-025-01820-2.

## Highlights

- Bifunctional atomic catalyst for dual environmental-energetic applications.
- Unprecedented catalytic performance: simultaneously 99% flowing-phase styrene degradation and 98% FE_CO_ at 1000 mA cm^−2^.
- Boron incorporation for accelerated electron transfer and optimized eCO_2_RR microenvironments.

**Supplementary Information:**

The online version contains supplementary material available at 10.1007/s40820-025-01820-2.

## Graphical abstract

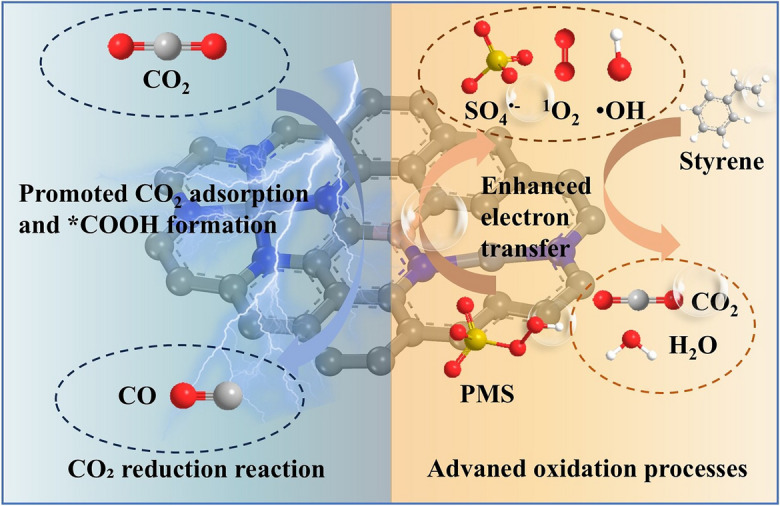

## Introduction

The dependence on fossil fuels leads to the generation of volatile organic pollutants (VOCs) and the accumulation of greenhouse gases [1, 2]. How to effectively degrade VOCs and further convert them into value-added chemical products. The innovative concept of “carbon closed-loop utilization” also provides new insights for simultaneously solving environmental problems and energy crises. The new advanced oxidation wet scrubbing process has been widely studied for VOCs removal due to its high efficiency, simple operation, and energy conservation [3, 4]. Different from the sequential batch reaction of the AOPs process for removing water pollutants, the continuous flow of VOCs passes through the liquid phase in the form of bubbles [5–7]. Therefore, highly active and stable catalysts are required for the efficient removal and deep mineralization of VOCs. Utilizing electrochemical CO_2_ reduction to convert into value-added chemicals is a valuable way to achieve carbon neutrality [8–10]. The important product CO can be applied to the Fischer–Tropsch synthesis process, and the selective generation of CO is also highly dependent on catalysts with high intrinsic activity [11–13]. In this regard, from the perspective of practical application, the development of bifunctional catalysts for converting waste gas into fuel is more attractive.

Recently, embedding atomically dispersed metal sites into carbon supports to form single-atom catalysts has attracted extensive attention from researchers due to its excellent atomic utilization efficiency and high selectivity [2, 14–16]. Carbonaceous materials with advantages such as large specific surface area and porous surface structures are regarded as ideal supports for constructing adsorption-catalysis materials [17, 18]. They are conducive to the rapid capture of gas molecules on the catalyst surface, which will greatly promote the adsorption of VOC gases and CO_2_ on the catalyst surface, as well as the contact and reaction between target VOCs and ROS [19]. The advanced oxidation process based on peroxymonosulfate (PMS) generates active species by breaking the O–O bond in PMS to attack pollutants. Meanwhile, the activation of PMS involves adsorption and interfacial electron transfer processes, and the electronic structure of metal atoms often affects the generation of radicals and the O–O bond breakage in PMS [20, 21]. The principle of the CO_2_ reduction process is that the catalyst can easily adsorb CO_2_ and firmly bind the COOH intermediate to effectively activate CO_2_, and CO has a weak interaction with the catalyst surface and is easy to desorb [22]. The reported M-N_4_ structure catalysts (Ni-NC and Fe-NC) have high energy barriers for the formation of the key intermediate product COOH and the desorption of CO during the CO_2_ reduction process, and the single metal site limits the activity of the catalyst [23, 24]. Research reports that introducing adjacent metal sites near single metal sites to expand the synthesis of dual-atom catalysts exhibits more excellent catalytic activity [25–27].

The charge density of atomic sites is related to coordination number and electronic structure, which in turn affects the catalytic performance. Lin et al. changed the coordination number of Fe-NxSACs and theoretically calculated that Fe-NxSACs with a lower coordination number had a stronger PMS adsorption affinity [28]. In addition, the intrinsic activity of the catalyst can be adjusted by introducing axial ligands and doping heteroatoms. Various heteroatoms such as sulfur (S) and phosphorus (P) reported in previous studies have been doped into the first or second coordination shells of the active center to adjust the coordination environment and thus improve the catalytic performance [29–32]. Recently, Chai et al. modified the iron single-atom catalysts (Fe-NC) through an electronic structure regulation strategy, introducing phosphorus (P) into the second coordination shell to enhance the activation of peroxymonosulfate (PMS), exhibiting an outstanding activity in the oxidation of bisphenol A [29]. Sun’s research team used wool keratin as a precursor and successfully synthesized a sulfur-bridged copper-nickel dual-atom site catalyst (Cu-S-Ni/SNC). The disulfide bonds in wool keratin provided the conditions for the one-step formation of S-bridged sites. A large current density of up to 550 mA cm^−2^ was achieved in the flow electrolytic cell [31]. In comparison, the role of electron-deficient boron in dual-atom catalytic sites remains to be investigated. It has a similar atomic radius to nitrogen atom, and there is an electronegativity difference. Therefore, it is expected that combining electron-deficient boron sites with the bimetallic atom coordination structure can achieve attractive Fenton-like and CO_2_ reduction performances. Meanwhile, Chitosan, a naturally occurring alkaline cationic polysaccharide, can be derived via deacetylation from chitin sources such as shrimp, crabs, crustacean insects, and plant cell walls [33, 34]. It offers numerous advantages, including abundant availability, renewability, and non-toxicity. The C_2_-NH_2_ group within the chitosan molecular chain serves as a CO_2_ recognition site, demonstrating a strong adsorption capacity for CO_2_. The presence of hydroxyl and amino groups within its structure imparts strong affinity, particularly for transition metals, enabling effective chelation and coordination with metal ions to form stable complexes [35, 36]. This characteristic also provides a foundation for the dispersion of metal active sites. Its excellent biocompatibility enables the modification of chitosan through both physical and chemical methods to generate materials such as activated carbon, which are extensively applied in fields such as CO_2_ reduction reactions.

Herein, this study designs an asymmetric bimetallic atomic site anchored on boron–nitrogen co-doped porous carbon catalyst (NiFe-BNC) by reasonably adjusting the coordination environment. Utilizing a chitosan-derived carbon substrate enriched with hydroxyl and amino groups, the inherent functional groups not only enhance metal-ion affinity to achieve homogeneous dispersion of atomic sites but also synergize with "boron electron bridge" constructed via secondary sintering, thereby cooperatively realizing significantly enhanced catalytic activity. In situ characterization and theoretical simulations demonstrate that boron incorporation: (i) enriched carbon substrate defects, optimizing PMS adsorption configurations to accelerate interfacial electron transfer; (ii) optimized the local microenvironment of catalyst in dynamic CO_2_ reduction process; (iii) strengthened CO_2_ adsorption lowers the energy barrier for *COOH intermediate formation and facilitates CO desorption, thereby dynamically suppressing hydrogen evolution reaction (HER). This work establishes a coordination-environment engineering paradigm for designing cost-effective dual-functional catalysts toward sustainable carbon recycling.

## Experimental Section

*Synthesis of NiFe-BNC*. 0.4 g of chitosan and 30 mg of urea were dissolved in 50 mL of ultrapure water, and then, 0.4 g of nickel chloride was added. The solution was stirred continuously until green solution was formed. 1.43 g of anhydrous ferric chloride was dissolved in 10 mL of water to form brown solution. The two solutions were then mixed and stirred at room temperature for 12 h. The mixture was then evaporated at 85 °C to remove the water, followed by continuous drying in a forced air-oven. The dried sample was placed in a tubular furnace and heated under an argon atmosphere at 750 °C for 4 h and then allowed to cool to room temperature. After washing with 6 M HNO_3_ and soaking, the sample was vacuum-dried. The dried powder (0.15 g) was mixed with a certain amount of boric acid (0, 0.015, and 0.025 g) and ground for 5 min. The mixture was then heated under an argon atmosphere at 900 °C for 2 h to obtain the NiFe-BNC. NiFe-BNC catalysts with different metal loading amounts were also prepared as control samples (where the molar amounts of Fe/Ni were 3/1 and 6/2). In addition, the preparation of NiFe-NC was similar to that of NiFe-BNC, except for the addition of the boric acid. The preparation of Ni–NC was similar to that of NiFe-NC, except for the addition of the Fe salt. The preparation of Fe-NC was similar to that of NiFe-NC, except for the addition of the Ni salt.

## Results and Discussion

### Catalyst Design and Characterizations

**Fig. 1:**
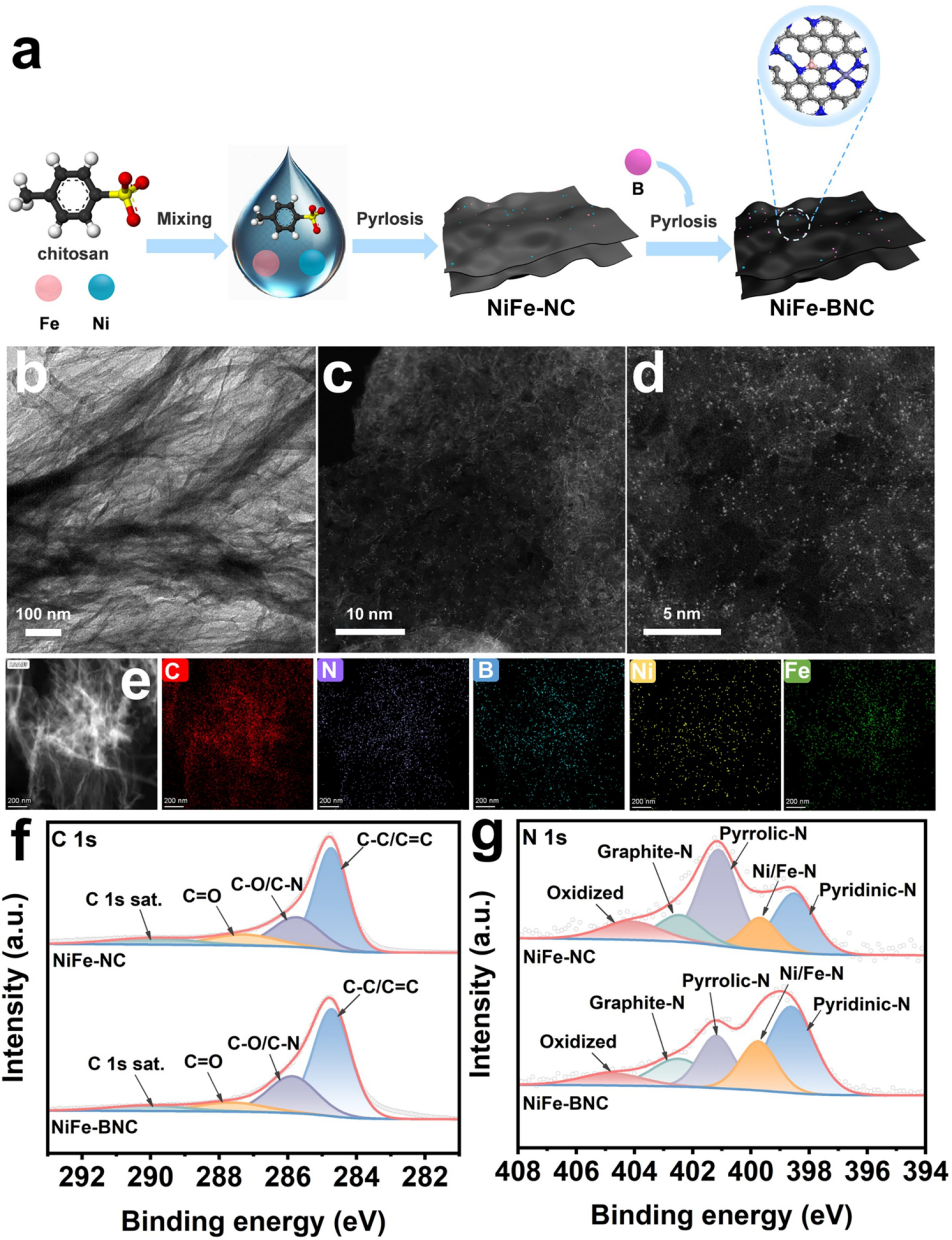
**a** Schematic preparation diagram of the catalysts. **b** TEM images, **c**–**d** AC-HAADF- STEM images and **e** EDS mapping of NiFe-BNC. XPS spectrum: **f** C 1*s*; **g** N 1*s*

As shown in Fig. 1a, chitosan rich in biofunctional groups was used as the substrate. It has a carbon skeleton containing amino functional groups. Meanwhile, the hydroxyl and amino groups in its structure endow it with strong affinity and good chelating ability for transition metals. It can coordinate with metal ions to form complexes, which helps to disperse metal active sites, and also has a good adsorption capacity for CO_2_ [16]. Chitosan served as the carbon and nitrogen sources for coordinating with Fe and Ni atoms, and boric acid was used as the B source. The NiFe-BNC catalyst was successfully synthesized through a two-step calcination strategy. For comparison, control samples of Ni-NC, Fe-NC, and NiFe-NC were also prepared using a similar strategy. Due to its excellent catalytic performance, NiFe-BNC was selected as a model catalyst for studying VOC degradation and CO_2_ conversion performance. As revealed from the transmission electron microscopy (TEM) image (Fig. 1b) and the scanning electron microscopy (SEM) images (Fig. S1), the chitosan-derived substrate exhibited an obvious porous structure without obvious aggregation of iron and nickel nanoparticles and clusters. The energy-dispersive X-ray spectroscopy (EDS) elemental mapping images show that the elements of Ni, Fe, N, B, and C are uniformly distributed in NiFe-BNC, and the iron element may be more densely distributed than the nickel element. Similarly, NiFe-NC also presents a uniform distribution of the elements Ni, Fe, N, and C (Fig. S2). The X-ray powder diffraction (XRD) pattern verifies the composition and structural evolution of the catalyst. As shown in Fig. S3, two diffraction peaks were observed at approximately 26°–44°, which are, respectively, attributed to the (002) and (101) crystal planes of graphite carbon [37, 38]. The aberration-corrected high-angle annular dark-field scanning transmission electron microscopy (AC-HAADF-STEM) was used to study the metal atom dispersion of NiFe-BNC. As shown in Fig. 1c, d, isolated bright spots are anchored on the nitrogen-doped porous carbon substrate. X-ray photoelectron spectroscopy (XPS) characterization was performed to determine the chemical states and electronic structure of the catalyst (Fig. 1f, g). The C 1*s* spectrum of NiFe-BNC shows peaks at 284.7, 285.9, 287.6, and 290.1 eV, which are attributed to C–C/C = C, C–O/C-N, C = O, and the C 1*s* satellite peak, respectively [39]. The N 1*s* spectrum shows peaks at 398.6, 399.7, 401.2, 402.5, and 404.8 eV, corresponding to pyridinic nitrogen, Ni/Fe–N, pyrrolic nitrogen, graphitic nitrogen, and oxidized nitrogen, respectively [40, 41]. Compared to NiFe-NC, the N 1*s* binding energy of NiFe-BNC exhibits a redshift due to the interactions induced by boron doping. Notably, the proportion of pyridinic nitrogen in NiFe-BNC (40.23%) increased compared to NiFe-NC (24.96%), while the proportion of pyrrolic nitrogen decreased in NiFe-BNC (18.81%) compared to NiFe-NC (29.63%). Meanwhile, the proportion of Fe/Ni–N species significantly increased (11.43 to 18.30%), indicating that boron atoms replaced the part of the pyrrolic nitrogen and optimized the substrate coordination environment. Additionally, the Fe–N content was enhanced due to carbon gasification mediated by the oxygen atoms in boric acid [42]. For the B 1*p* spectrum of NiFe-BNC, peaks at 191.9 and 192.9 eV correspond to BC_2_O and BCO_2_, respectively (Fig. S5) [43, 44].

**Fig. 2:**
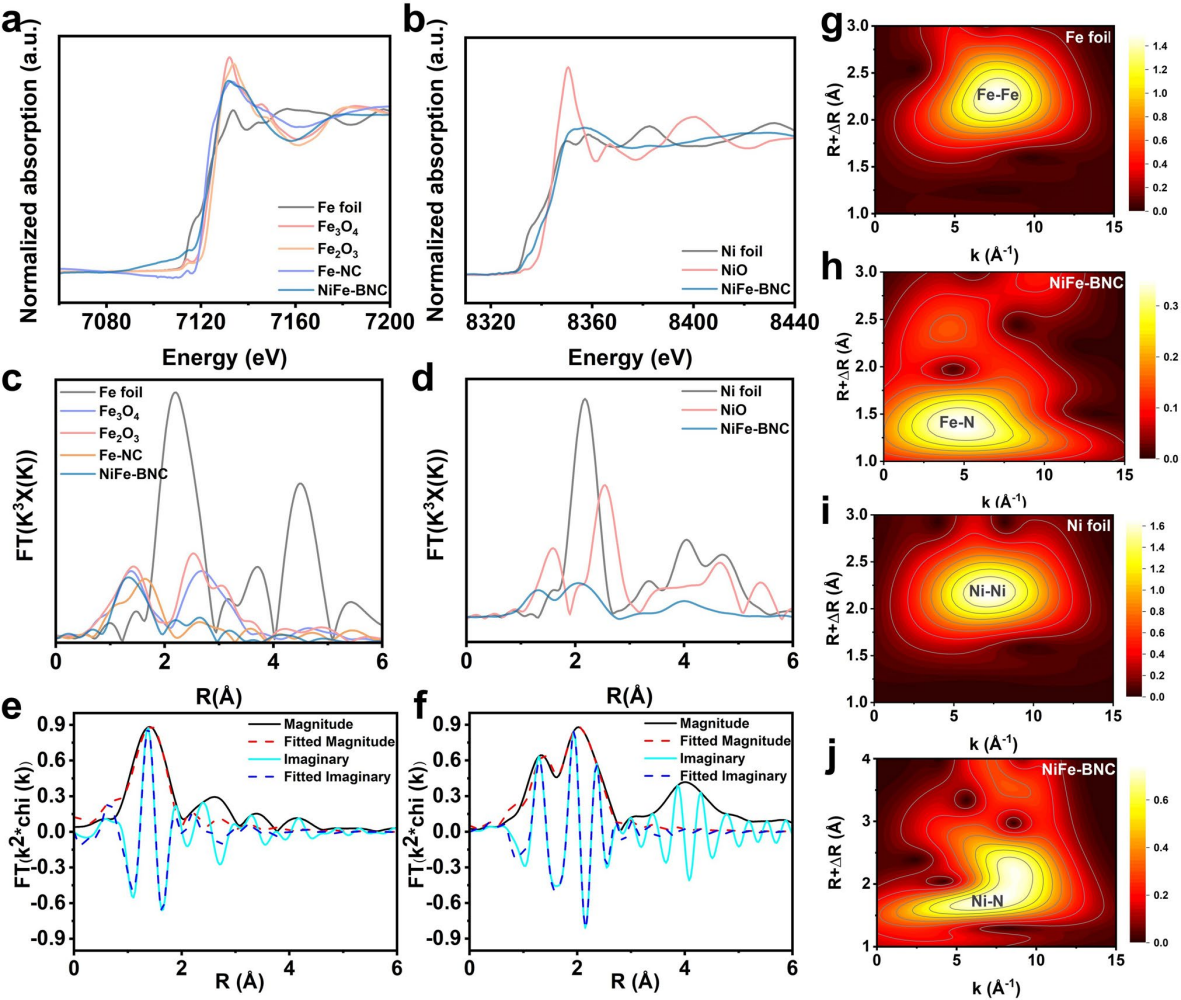
**a** XANES spectra of the Fe K-edge. **b** XANES spectra of the Ni K-edge. **c** Fe K-edge EXAFS spectra comparison of Fe foil, Fe_3_O_4_, Fe_2_O_3_, Fe-NC and NiFe-BNC. **d** Ni K-edge EXAFS spectra comparison of Ni foil, NiO, and NiFe-BNC. **e**–**f** Experimental and fitting EXAFS curves of NiFe-BNC in R space. **g**–**j** WT-EXAFS spectra at the Fe K-edge and Ni K-edge of Fe foil, Ni foil and NiFe-BNC

The functional group structure of the catalyst was investigated using Fourier transform infrared (FTIR) spectroscopy (Fig. S6b). Specifically, the peak at 3422 cm^−1^ corresponds to the O–H stretching vibration, while the peaks around 1410–1580 cm^−1^ are attributed to C–O and C=C groups, respectively. The weak peak at 1166 cm^−1^ is associated with the C–B/B–O bond, confirming the successful doping of boron. Raman spectroscopy was employed to explore the carbon structural defects in the catalyst. Two characteristic peaks corresponded to the D peak (carbon defects) and the G peak (graphitization degree of *sp*^2^-hybridized carbon) (Fig. S6a). Notably, the I_D_/I_G_ ratio of NiFe-BNC (1.02) was higher than that of NiFe-NC (0.973), confirming that boron doping leads to more defects and disorder on the nitrogen-doped carbon substrate.

The coordination environment and electronic structure of Fe/Ni active sites in the catalysts were systematically investigated through X-ray absorption near-edge structure (XANES) and extended X-ray absorption fine structure (EXAFS) analyses. Figure 2a presents the Fe K-edge XANES spectra of Fe-NC and NiFe-BNC catalysts, with reference samples of Fe foil, Fe_2_O_3_, and Fe_3_O_4_. The absorption edge position of Fe–NC lies between those of Fe_2_O_3_ (+ 3 oxidation state) and metallic Fe foil, while NiFe-BNC exhibits an intermediate edge position between Fe-NC and Fe foil. This shift was attributed to the modulation effects of the coordination environment and B dopant on the electronic structure. Similarly, Ni K-edge XANES spectra (Fig. 2b) reveal that the absorption edge of NiFe-BNC is positioned between Ni foil (0) and NiO (+ 2), indicating a positively charged Ni species. These results collectively demonstrate that both Fe and Ni in NiFe-BNC exist in oxidation states ranging from 0 to + 2, with their electronic configurations being mutually influenced through Fe–Ni interactions and B-doping effects. The local coordination environment of metal sites was further elucidated by K-edge EXAFS spectroscopy. As shown in Fig. 2c, both Fe-NC and NiFe-BNC display dominant peaks at approximately 1.64 and 1.33 Å in Fe K-edge EXAFS spectra, corresponding to Fe–N coordination. Notably absent are Fe–Fe scattering signals characteristic of metallic Fe (Fe foil) or iron oxides (Fe_3_O_4_, Fe_2_O_3_), confirming the atomic dispersion of Fe in NiFe-BNC. The Ni K-edge EXAFS spectrum of NiFe-BNC (Fig. 2d) exhibits a prominent Ni–N coordination peak at − 1.32 Å, accompanied by a distinct Ni–Ni scattering peak at 2.06 Å, indicative of partial Ni clustering. Wavelet transform (WT) contour plots (Fig. 2g–j) visually confirm the coexistence of atomically dispersed Fe and Ni atoms with Ni clusters in NiFe-BNC. Least-square EXAFS fitting was performed to quantify the coordination parameters of NiFe-BNC (Fig. 2e, f). An excellent agreement between experimental data and fitted curves validates the structural model (detailed fitting parameters in Table S1). The fitting results confirm Fe–N and Ni–N coordination configurations without detectable Fe–Ni bonding. These findings substantiate the dual-atom synergistic mechanism in NiFe-BNC, where Fe and Ni atoms are stabilized through metal-N coordination within the nitrogen-doped carbon framework, while maintaining distinct electronic properties through heteroatomic interactions and B-mediated electronic regulation.

### Evaluation of VOC Degradation Performance

**Fig. 3:**
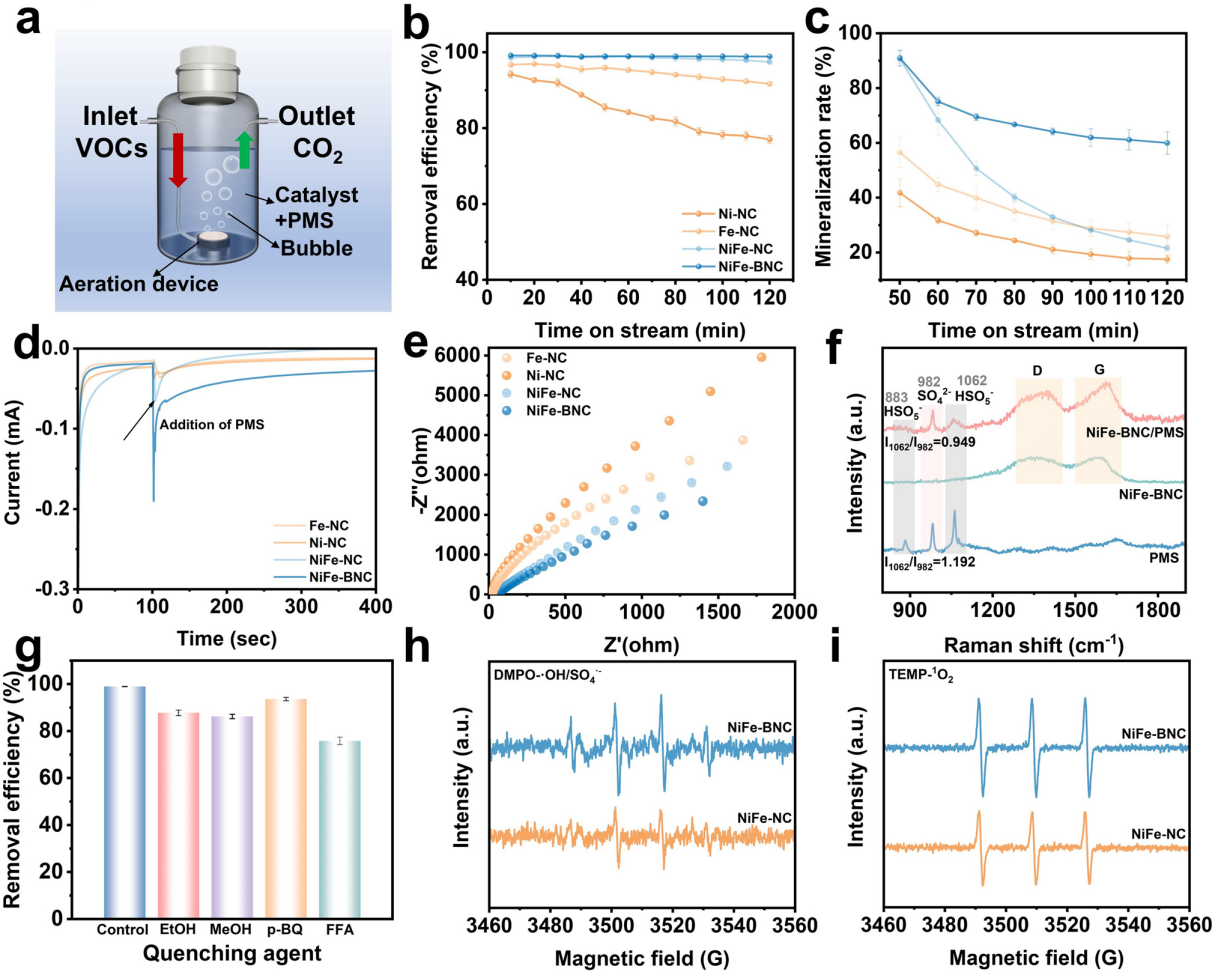
**a** Schematic Experimental setup of gaseous styrene degradation in the AOP wet scrubber. **b** Removal efficiency of styrene in various catalytic systems. **c** Corresponding outlet CO_2_ concentration and mineralization rate. **d** Current responses after the sequential injection of PMS. **e** Electrochemical impedance spectra of different catalysts. **f** In situ Raman spectra of PMS, NiFe-BNC, NiFe-BNC/PMS. **g** Effect of various radical scavengers on the styrene degradation performance. **h**–**i** EPR spectra in the catalyst/PMS system

Catalytic degradation experiments of gaseous VOCs were conducted using the designed advanced oxidation wet scrubbing process (Fig. 3a). Styrene was used as the model VOC to systematically investigate the catalytic performance of different catalysts in activating PMS for the degradation of styrene in the liquid phase. As shown in Fig. 3b, during the 2 h continuous reaction, the Ni-NC/PMS system initially exhibited a degradation efficiency of 93.53% in the first 10 min, which gradually decreased to 77.8% after 2 h; the Fe-NC/PMS system decreased from an initial 96.3 to 91.3%, and the NiFe-NC/PMS system also showed a decreasing trend in efficiency over the 2 h reaction period. The introduction of B atoms resulted in an outstanding stability efficiency of 99.02% for the NiFe-BNC/PMS catalytic system. As far as we know is much better than other currently reported research results. However, the CO_2_ content at the outlet gradually decreased over time in the continuous flow system. This is because PMS is gradually consumed as the reaction proceeds and the relatively insufficient reactive oxygen species (ROS) tend to react with the gaseous styrene first, rather than participating in the mineralization process [3]. It is noteworthy that when the reaction reaches 120 min, the mineralization rate of NiFe-BNC remained above 60%, which is significantly higher than that of NiFe-NC (Fig. 3c). Furthermore, in addition to exhibiting excellent styrene removal performance during the first 120 min of the continuous flow catalytic reaction, there was no significant decrease in efficiency after five consecutive cycles, and the CO_2_ mineralization rate remained at 50% (Fig. S23). It can be reasonably inferred that a synergistic effect exists between the adsorption of the porous carbon substrate and the catalytic active sites. Styrene first adsorbs onto the catalyst surface, and then, the catalyst activates PMS to generate ROS for oxidation. The introduction of electron-deficient boron regulates the atomic structure, enhancing its utilization through adsorption and catalytic effects, thereby effectively synergizing to enhance VOC removal. After the AOP reaction, the NiFe-BNC catalyst retained its original morphological structure (Fig. S7), indicating the high stability of the catalyst. In summary, based on the structural characteristics and catalytic performance, the improvement of NiFe-BNC compared to NiFe-NC in catalytic efficiency may be attributed to the introduction of boron, which created more defects on the carbon substrate, thereby promoting ROS generation. Additionally, the boron atoms regulated the formation of more Fe/Ni–N single-atom active sites, optimizing the PMS adsorption configuration.

Based on the current results and analysis, it can be reasonably inferred that the generation of ^1^O_2_ originates from PMS oxidation [21]. Charge transfer in different catalyst/PMS systems was monitored by recording the amperometric I–t curve (Fig. 3d). Upon the injection of PMS, a negative current was immediately observed. In contrast, NiFe-BNC exhibited a distinct negative current response. These observations suggest that, during the PMS injection phase, electrons can transfer from the catalyst to PMS, with NiFe-BNC transferring more electrons to PMS [45, 46]. This change in electron transfer leads to the oxidation of PMS and the formation of ^1^O_2_. Electrochemical impedance spectroscopy (EIS) was further used to measure the charge transfer resistance on the surface of different catalysts (Fig. 3e). Compared to Ni-NC, Fe-NC, and NiFe-NC, NiFe-BNC exhibited more efficient charge transfer at the interface [47].

To gain a deeper understanding of the electron transfer process between NiFe-BNC and PMS, we conducted in situ Raman spectroscopy analysis (Fig. 3f). Three distinct peaks were observed in the initial PMS solution. The peaks at 883, 982, and 1062 cm^−1^ correspond to the stretching vibration of the O–O bond in HSO_5_^−^, the symmetric stretching vibration of the S = O bond in SO_4_^2−^, and the stretching vibration of SO_3_ in HSO_5_^−^, respectively. After adding NiFe-BNC, the peaks of O–O and SO_3_ sharply decreased within 1 min, indicating that PMS was rapidly activated. Notably, the characteristic vibration peak of SO_3_ exhibited a slight blue shift [48]. The blue shift typically results from a decrease in electron density, suggesting that electrons transferred from PMS to the catalyst, and PMS tends to convert to SO_5_^•−^, which then reacts with water to generate ^1^O_2_ [49]. The electron transfer process was further studied using linear sweep voltammetry (LSV) analysis (Fig. S8). After adding PMS and styrene, a significant increase in the current on the electrode was observed, indicating that electrons transferred from PMS to NiFe-BNC [50]. This further validated the electron transfer process observed in the in situ Raman spectroscopy.

To elucidate the main reactive species involved in VOC degradation in the NiFe-BNC/PMS system, ethanol (EtOH, a scavenger of SO_4_^•−^ and •OH radicals), methanol (MeOH, a quencher of •OH radicals), para-benzoquinone (p-BQ, a quencher of O_2_^•−^ radicals), and furfural alcohol (FFA, a scavenger of ^1^O_2_ radicals) were used to determine the contribution of reactive oxygen species to VOC degradation (Fig. 3g) [51]. Upon the addition of ethanol, the styrene degradation rate decreased to 86% within the first 10 min, and the characteristic peak of benzene appeared. Over time, the peak area gradually increased, and quantitative measurements revealed a slow increase in benzene concentration (Fig. S20a). Similarly, the styrene degradation rate dropped to 84.7% after 10 min when methanol was added, and the characteristic benzene peak appeared after 30 min (Fig. S20b). This peak slowly intensified as the reaction time increased. The addition of para-benzoquinone had no significant impact on the styrene removal rate but affected the outlet CO_2_ concentration. After adding furfural alcohol, the styrene concentration dropped to 76.7% in the first 10 min, while the outlet CO_2_ concentration was severely affected, reaching only 10.6%. This indicates that sulfate radicals (SO_4_^•−^) and hydroxyl radicals (•OH) primarily contribute to the initial oxidation of styrene, converting it into intermediate products such as benzene. Meanwhile, singlet oxygen (^1^O_2_) and superoxide radicals (O_2_^•−^) play a role in the ring-opening process of the benzene ring and subsequent mineralization. Furthermore, the main gaseous and aqueous intermediate products from the VOCs reaction process were analyzed using gas chromatography–mass spectrometry (GC–MS). In the gas-phase products of the NiFe-BNC/PMS system, CO_2_ and nitrogen were detected, indicating effective removal and mineralization of styrene, thus preventing secondary air pollution caused by by-product formation and emissions. In the liquid-phase products, benzyl alcohol (m/z 108) and short-chain compounds were detected, including acetone (m/z 59), ethylene glycol (m/z 63), butanal (m/z 73), and 1,2-propanediol (m/z 77). Based on these results, a possible degradation pathway for styrene is proposed (Fig. S20c).

In the advanced oxidation wet scrubbing process, VOCs are introduced into the liquid phase via an aeration device, and a significant amount of molecular oxygen may potentially participate in the catalytic oxidation of VOCs. We conducted degradation experiments under anaerobic conditions to exclude this effect, further confirming that ^1^O_2_ primarily originates from the decomposition of PMS. Subsequently, electron paramagnetic resonance (EPR) spectroscopy was employed to verify the results of the quenching experiments, using 5,5-dimethyl-1-pyrroline N-oxide (DMPO) and 2,2,6,6-tetramethylpiperidine (TEMP) as spin traps. Characteristic peaks corresponding to SO_4_^•−^, •OH, ^1^O_2_, and O_2_^•−^ radicals were observed in both the NiFe-NC/PMS and NiFe-BNC/PMS systems (Figs. 3h, i and S9). However, the radical intensity in the NiFe-BNC/PMS system was significantly higher than in the NiFe-NC/PMS system, indicating that the introduction of boron atoms increased the carbon substrate defects, allowing for the adsorption of more PMS molecules onto the catalyst surface. This optimized the content of pyridine nitrogen and pyrrole nitrogen, increased the FeNi/N content and greatly facilitated the reaction between the catalyst and PMS, leading to the generation of more radicals and a significant enhancement in PMS utilization.

### Enhanced CO_2_ Electroreduction with NiFe-BNC Catalyst

**Fig. 4:**
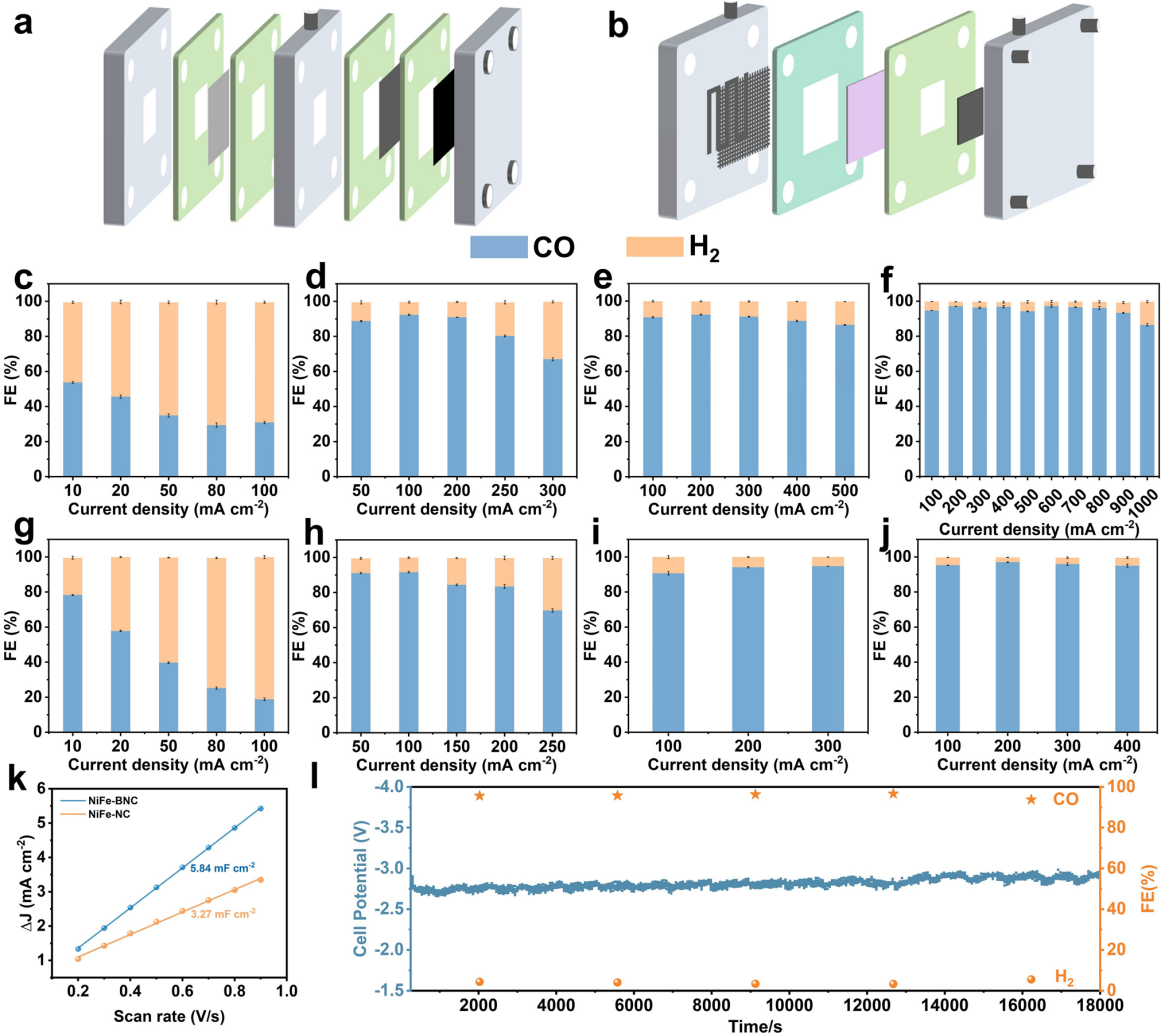
ECO_2_RR performance evaluations of different catalysts in flow-cell and MEA electrolyzers.. **a**–**b** Schematic diagram of the flow-cell electrolyzer and the MEA electrolyzer. **c** FE_CO_ of Fe-NC at different current densities in flow-cell and g MEA. **d** FE_CO_ of Ni-NC at different current densities in flow cell and **h** MEA. **e** FE_CO_ of NiFe-NC at different current densities in flow cell and **i** MEA. **f** FE_CO_ of NiFe-BNC at different current densities in flow cell and **j** MEA. **k** ECSAs of NiFe-NC and NiFe-BNC. **l** Stability test of NiFe-BNC in the MEA electrolyzers

The CO_2_ conversion performance of the catalyst was evaluated in a flow electrolyzer and a membrane electrode assembly (MEA) electrolyzer (Figs. 4a, b and S10, S11). The gaseous and liquid phase products were quantitatively analyzed using gas chromatography (GC) and ^1^H nuclear magnetic resonance (^1^H NMR) spectroscopy (Fig. S12). The results showed that carbon monoxide (CO) and hydrogen (H_2_) were the two main products, with no liquid phase products generated. As shown in Fig. S13, the linear sweep voltammetry (LSV) results of the catalyst indicated that the current density of NiFe-BNC in the CO_2_-saturated electrolyte was significantly higher than that of other catalysts, and also notably higher than the current density in Ar-saturated electrolyte. This suggests that the cathodic current primarily originates from CO_2_ reduction, with the reaction favoring eCO_2_RR rather than the HER [52]. To evaluate the CO_2_ catalytic performance of different catalysts, the Faradaic efficiency (FE) of H_2_ and CO production was measured across various current densities using a flow electrolyzer. The hydrogen FE for Fe-NC reached 68.17% at 100 mA cm^−2^, while the Ni-NC electrode showed an increase in FE from 8.94% at 200 mA cm^−2^ to 32.32% at 300 mA cm^−2^ (Fig. 4c–d).

The NiFe-BNC electrode exhibited relatively stable and low HER activity, with a minimum FE of 2.08% at 600 mA cm^−2^, gradually increasing to 13.7% at 1000 mA cm^−2^ (Fig. 4f). In contrast, the hydrogen FE for the NiFe-NC electrode increased from 10.96% at 300 mA cm^−2^ to 13.21% at 500 mA cm^−2^ (Fig. 4e). NiFe-BNC exhibited superior performance compared to NiFe-NC after the introduction of boron atoms, with a current density significantly higher than industrial levels (1000 mA cm^−2^), which is 3.3 times, 10 times, and 2 times greater than Ni-NC, Fe-NC, and NiFe-NC, respectively. Moreover, NiFe-BNC-0.015 and NiFe-BNC-0.025 catalysts with different boron doping levels were prepared for CO_2_ reduction performance studies. The results indicated that the maximum current densities of NiFe-BNC-0.015 (1000 mA cm^−2^) and NiFe-BNC-0.025 (500 mA cm^−2^) were all higher than those of Ni-NC and Fe-NC (Fig. S16). Excessive boron doping content accelerates the hydrogen evolution reaction. The results in Fig. S17b–d showed that NiFe3-BNC exhibited a more stable styrene degradation rate and superior CO_2_ reduction performance compared to NiFe1-BNC and NiFe2-BNC, indicating that lower metal loading limited the active sites, thus hindering the catalytic reaction. The catalytic activity and selectivity of this catalyst surpass those of most bimetallic nickel-based catalysts reported in the literature (Tables S2 and S3). This performance difference highlights the effectiveness of electron-deficient boron in the NiFe dual-atom configuration, especially under high current density conditions, where competition with the HER typically intensifies. Furthermore, the membrane–electrode assembly (MEA) electrolyzer was used to evaluate the stability of different catalysts under industrial current density conditions to reduce solution resistance and improve the energy efficiency of the electrolyzer. The MEA electrolyzer was maintained at 303.15 K using a temperature control device to accurately assess the catalytic performance. Fe-NC only achieved 18.3% CO Faraday efficiency at 100 mA cm^−2^ current density, while the FE_CO_ decreases to 69.1% at 250 mA cm^−2^ for Ni-NC (Fig. 4g, h). Applying a higher cell voltage resulted in an increased current density, reaching 400 mA cm^−2^, with a CO selectivity of 97.16% (Fig. 4j). In contrast, the NiFe-NC catalyst exhibited a maximum FE_CO_ of 94.73 mA cm^−2^ at 300 mA cm^−2^ (Fig. 4i). Additionally, to investigate the synergistic effect of Ni and Fe in NiFe-NC, the electrocatalytic CO_2_RR performance of catalysts prepared by physically mixing Ni-NC and Fe-NC was evaluated, with the metal loading matching that of NiFe-NC. We further calculated the electrochemical active surface areas (ECSAs) of different catalysts (Fig. 4k). The CdI value of NiFe-BNC (5.84 mF cm^−2^) is significantly higher than that of NiFe-NC (3.27 mF cm^−2^), indicating that NiFe-BNC has superior intrinsic activity in eCO_2_RR compared to NiFe-NC [53]. Effective CO_2_-to-CO conversion also requires an assessment of its stability. Therefore, the stability of the NiFe-BNC catalyst was tested at an optimized current density of 100 mA cm^−2^. Regular gas chromatography measurements indicated that the catalyst maintained a high CO Faradaic efficiency over continuous electrolysis (Fig. 4l). The CO_2_ temperature-programmed desorption (CO_2_-TPD) test indicated that the NiFe-BNC catalyst exhibited significantly enhanced CO_2_ chemisorption capacity compared to the NiFe-NC catalyst, with the CO_2_ adsorption capacity of the former being 6 times that of the latter (Fig. S19). Electrochemical impedance spectroscopy (EIS) data show that the charge transfer resistance of NiFe-BNC is lower than that of NiFe-NC, Ni-NC, and Fe-NC, indicating that NiFe-BNC exhibits faster charge transfer capability [52]. As can be seen from Figs. 4e and S15, NiFe-NC > Fe-NC + Ni-NC, which indicated that, in addition to the isolated metal active sites, the other active contributors of the prepared NiFe-NC must be attributed to the synergistic effect between the Fe and Ni atom pairs.

### Description of Catalytic Mechanism

**Fig. 5:**
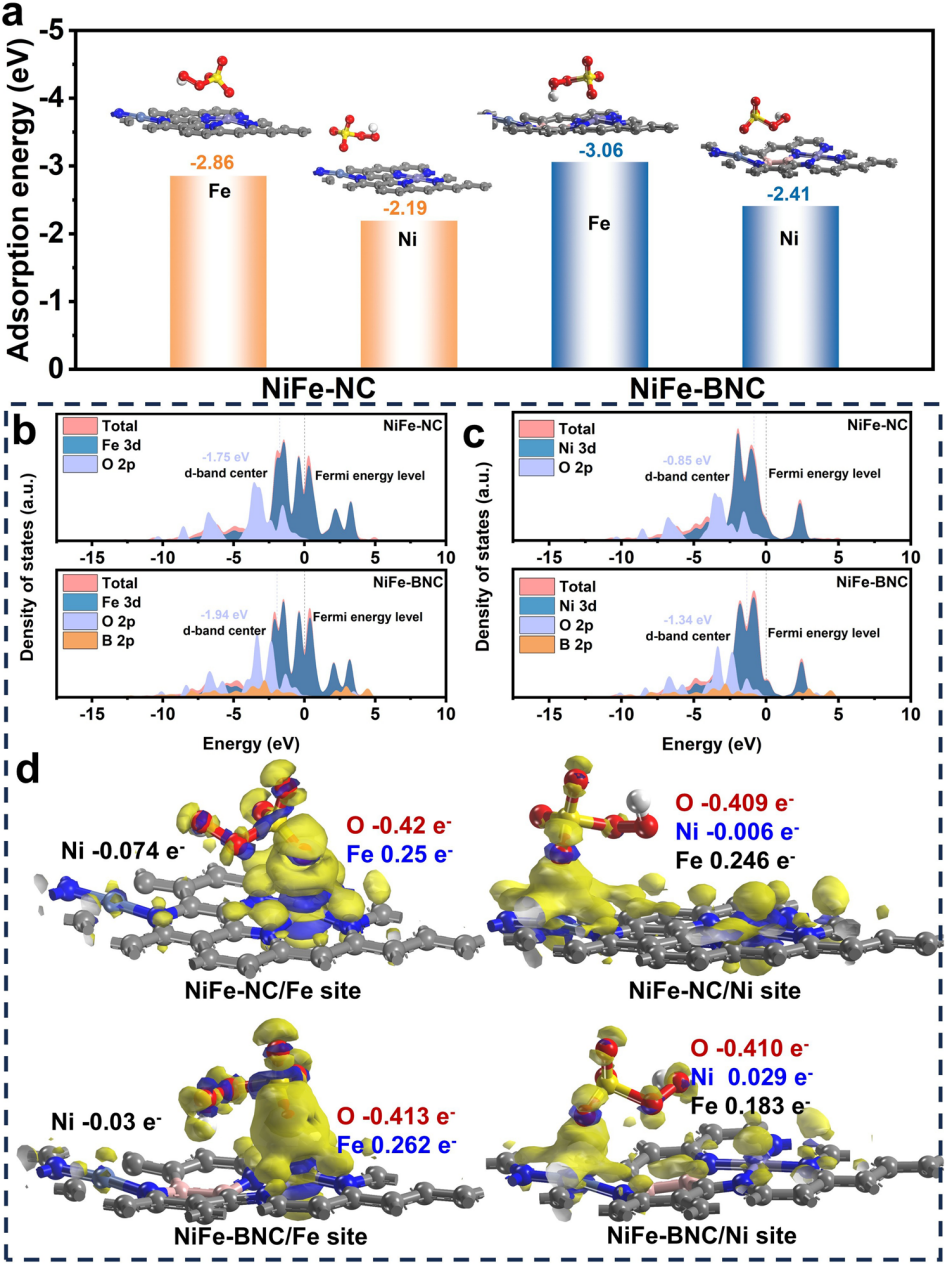
**a** PMS adsorption energy of NiFe-NC and NiFe-BNC catalysts. **b**–**c** PDOS diagram of NiFe-BNC and NiFe-NC adsorbing PMS. **d** Charge density maps of adsorption of PMS at different metal sites of NiFe-NC and NiFe-BNC

To further elucidate the reaction mechanism of the NiFe-BNC catalyst and investigate the influence of B-modulated coordination environments in bimetallic sites on catalytic pathways, systematic density functional theory (DFT) calculations were performed. Structural models of the catalysts were constructed based on synchrotron radiation results and optimized to simulate potential active sites under realistic catalytic conditions. Distinct peroxymonosulfate (PMS) adsorption configurations involving bonding with different oxygen atoms lead to divergent PMS activation pathways and reactive species generation. We thus analyzed the adsorption energies of O1 and O2 atoms in PMS adsorption configurations, revealing that the NiFe-NC catalyst exhibits stable adsorption selectivity toward the O1 site in PMS (Fig. S27). Consequently, the effects of NiFe-NC and NiFe-BNC on O1-site interactions were further explored. The adsorption energies of PMS on Fe sites in NiFe-NC and NiFe-BNC were calculated as − 2.86 and − 3.06 eV, respectively, while those on Ni sites were − 2.19 and − 2.41 eV, respectively (Fig. 5a). The enhanced adsorption energies in NiFe-BNC demonstrate that B incorporation facilitates PMS adsorption and decomposition to generate reactive oxygen species.

Projected density of states (PDOS) analysis was employed to decipher the catalytic mechanism through which B atoms optimize the microenvironment of Fe–Ni dual-atomic sites. PDOS profiles of Fe 3*d*, Ni 3*d*, and O 2*p* orbitals were examined for PMS-adsorbed NiFe-NC and NiFe-BNC systems. As shown in Fig. 5b, c, significant orbital overlaps between Fe 3*d*/Ni 3*d*, O 2*p* and B 2*p* orbitals were observed upon PMS adsorption, confirming robust chemical interactions between the catalyst and PMS. Notably, the d-band centers of both Fe (− 1.94 vs. − 1.75 eV) and Ni (− 1.34 vs. − 0.85 eV) in NiFe-BNC exhibited distinct downshifts compared to NiFe-NC, suggesting that B doping weakens adsorption strength and potentially optimizes intermediate desorption [54].

Charge transfer dynamics between the catalysts and PMS were investigated through differential charge density analysis. Figure 5d clearly displays pronounced electron density redistribution at the catalyst–PMS interface, where blue and yellow isosurfaces denote electron accumulation and depletion, respectively. Compared to NiFe-NC, both Fe and Ni sites in NiFe-BNC demonstrated enhanced charge transfer magnitudes, evidencing superior electron transfer capabilities. These findings collectively verify that B atomic doping effectively enhances electronic transfer efficiency, thereby rationalizing the improved catalytic performance of NiFe-BNC.

**Fig. 6:**
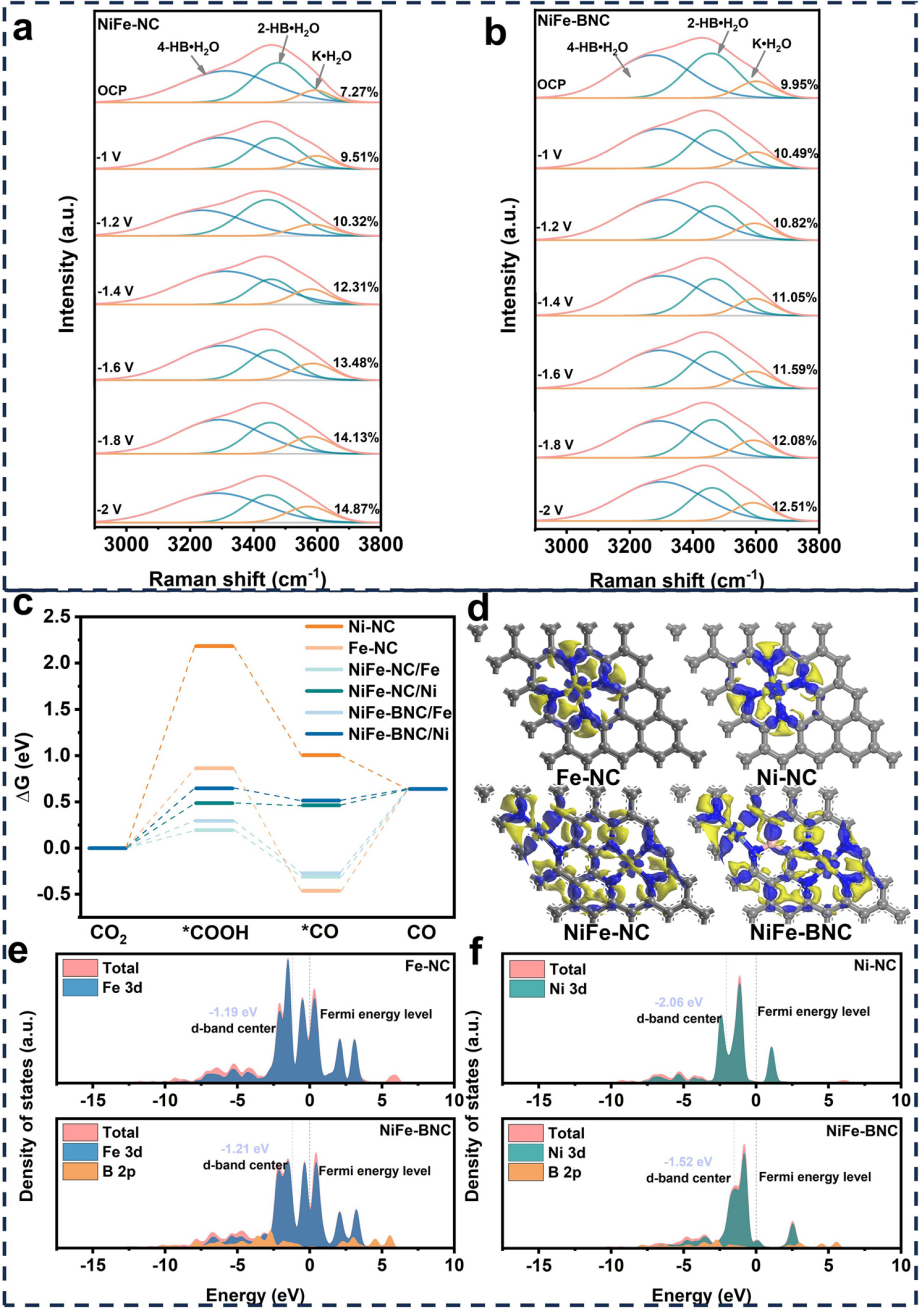
**a**–**b** In situ Raman of NiFe-NC and NiFe-BNC during CO_2_ reduction: interfacial water structure at different potentials; **c** Gibbs free energy diagram of CO_2_ reduction at different catalyst metal sites; **d** charge density maps of metal sites of Fe-NC, Ni-NC, NiFe-NC, and NiFe-BNC catalysts; **e**–**f** PDOS of Fe and Ni three-dimensional orbits corresponding to Fe-NC, Ni-NC, and NiFe-BNC structures

To gain deeper insights into the reactive microenvironment of catalysts, we performed in situ Raman spectroscopy to elucidate the interfacial water structures of NiFe-NC and NiFe-BNC catalysts at varying potentials. Both catalysts exhibited characteristic O–H stretching modes of interfacial water (Fig. 6a, b). These spectral features were deconvoluted into three distinct components corresponding to different hydrogen-bonding configurations: tetra-coordinated hydrogen-bonded water (4-HB·H_2_O) at − 3200 cm^−1^, di-coordinated hydrogen-bonded water (2-HB·H_2_O) at − 3400 cm^−1^, and K⁺-coordinated water (K·H_2_O) at − 3600 cm^−1^. The localized K⁺ concentration plays a critical role in suppressing the HER [55]. Quantitative analysis revealed a general increase in K·H_2_O percentage under higher negative potentials, indicative of weakened hydrogen bonding and enhanced ^*^H coverage. Specifically, the K·H_2_O contribution on NiFe-NC surfaces increased from 7.27 to 14.87% as the applied potential shifted from 0 to − 2 V, whereas NiFe-BNC exhibited only a marginal increase from 9.95 to 12.51% [56]. This reduced K·H_2_O population in NiFe-BNC facilitates more favorable HER kinetics, suggesting stronger water dissociation and *H adsorption activity in NiFe-NC compared to NiFe-BNC.

DFT calculations were employed to elucidate the enhanced eCO_2_RR activity of NiFe-BNC. The CO_2_-to-CO conversion involves two critical proton-coupled electron transfer steps with ^*^COOH and CO as key intermediates. Initially, CO_2_ adsorption occurs on the catalyst surface to form ^*^COOH, followed by protonation to generate ^*^CO and H_2_O, with subsequent desorption of gaseous CO [57]. Gibbs free energy calculations for each elementary step were performed using structural models derived from EXAFS fitting results. As shown in Fig. 6c, the energy barriers for CO_2_ hydrogenation (CO_2_ → ^*^COOH) on Fe-NC and Ni-NC were 0.18 eV and 2.18 eV, respectively. The substantially higher formation energy of ^*^COOH on Ni-NC aligns with previous reports identifying this step as the rate-determining process for Ni-based single-atom catalysts [31]. In contrast, Fe-NC exhibited a rate-limiting step in CO desorption due to its stronger CO adsorption. Remarkably, the dual-atomic Fe–Ni sites in NiFe-BNC demonstrated optimized reaction pathways compared to their single-atom counterparts. The ^*^COOH formation energy on Ni sites decreased significantly from 2.18 (Ni-NC) to 0.65 eV (NiFe-BNC), while the CO adsorption energy on Fe sites weakened from − 0.46 eV (Fe-NC) to − 0.27 eV (NiFe-BNC). Both the ^*^COOH formation and CO desorption barriers in NiFe-BNC were lower than those in NiFe-NC, corroborating the superior catalytic activity of NiFe-BNC.

Further comparative analysis of activation free energies revealed that Fe sites in NiFe-BNC exhibited lower energy barriers than Ni sites during the hydrogenation of activated CO_2_. These findings suggested that electronic structure modulation through intrinsic electron transfer between Fe and Ni dual-atomic sites, facilitated by boron doping, optimized adsorption strength of reaction intermediates while maintaining high CO selectivity. Charge density mapping (Fig. 6d) revealed asymmetric charge distribution in NiFe-BNC, with significant electron transfer observed among Fe–Ni, Fe-B, and Ni–B atoms. Based on the Mulliken charge distribution analysis from DFT calculations (Fig. S29a, b), it was observed that after boron doping, B replaced C in the structure. Since B had one fewer valence electron than C, it disrupted the C–C π bonds and created a positively charged center due to its lower electronegativity compared to C, thereby altering the local charge transfer on the catalyst surface. To reveal the influence of p-d orbital hybridization on the active sites, the partial density of states (PDOS) of the Ni, Fe, and B atoms in the NiFe-BNC model were calculated (Fig. 6e–f). The results indicated significant orbital overlap between the B atom and both Fe and Ni atoms. By measuring the Fe/Ni–N coordination bond length and bond angle in the structural model, it was found that the Fe/Ni–N bond length increased after B doping, and the Ni–N coordination bond angle decreased from 176.32° to 174.48° (Fig. S29c, d). This was due to the increased charge on the carbon atoms surrounding B and the decreased charge on the nitrogen (N) atoms, which weakened the bonding between N and Fe/Ni. As a result, the d-band centers of Fe and Ni rose, enhancing the adsorption of oxygen-containing intermediates on the metal centers, which further promoted the CO_2_ reduction reaction. In the Raman spectra shown in Fig. S6, the distortion introduced by boron doping led to an increase in the intensity of the D peak (defect-related peak) and a shift of the G peak to lower wavenumbers. This reflected changes in the lattice strain and electronic structure of the catalyst surface [58], further confirming the DFT calculation results. This underscores the pivotal role of boron as an electronic "bridge" mediating interfacial charge redistribution. Additional calculations of d-band centers showed that both Ni and Fe 3*d* orbitals in NiFe-BNC underwent distinct shifts compared to their single-metal counterparts. The Ni sites exhibited higher density of states near the Fermi level than Fe sites, while the overall d-band center of NiFe-BNC shifted closer to the Fermi level compared to Ni-NC, indicating enhanced electron transfer kinetics. These comprehensive theoretical analyses elucidate the fundamental mechanism behind the exceptional CO_2_RR performance of NiFe-BNC catalysts.

## Conclusions

In summary, we have innovatively developed a novel dual-atom functional catalyst supported on chitosan-derived nitrogen-doped carbon. The complementary synergistic effect is achieved through the interaction between adjacent metal species and the substrate "boron electron bridge", exhibiting extraordinary catalytic performance for VOC degradation (achieving a 99% styrene degradation rate and a stable CO_2_ conversion rate of over 60% in a 2 h continuous flow reaction) and CO_2_ reduction reaction (achieving a large current density of 1 A cm^−2^ and a CO Faraday efficiency of 98%). Advanced experimental characterization and in-depth theoretical exploration were conducted to analyze the coordination structure of catalysts, revealing the underlying reasons for its exceptional performance. The introduction of boron atoms not only induces defects in the carbon substrate but also optimizes the nitrogen content in various forms on the carbon support. This accelerates the electron transfer between the catalyst and PMS, while simultaneously promoting CO_2_ adsorption and the formation of ^*^COOH, significantly lowering the desorption energy barrier for CO. It provides feasible and important insights for realizing the recycling of waste gas into carbon resources.

## Supplementary Information

Below is the link to the electronic supplementary material.

Supplementary file1 (DOC 10211 KB)
