# Supplementary material for: B-Bridge Regulated Asymmetric Dual-Atomic Catalysts for Synergistically Enhanced Styrene Mineralization and CO2 Reduction
Source: Nanomicro Lett. 2025 Jun 23;17:304. doi: 10.1007/s40820-025-01820-2 (PMC12185818; doi:10.1007/s40820-025-01820-2)
Supplement: Supplementary file 1 — Supplementary file1 (DOC 10211 KB) [file 40820_2025_1820_MOESM1_ESM.doc]

Supporting Information for

**B-Bridge Regulated Asymmetric Dual-Atomic Catalysts for Synergistically Enhanced Styrene Mineralization and CO_2_ Reduction**

Xiai Zhang^1#^, Zhongshuang Xu^1#^, Xinwei Zhang^1^, Jingquan Wang^1^, Dan Liu^1^, Huanran Miao^1^, Tong Wang^1^, Zhimao Yang^1^, Qikui Fan^1^, Chuncai Kong^1^*

^1^MOE Key Laboratory for Non-Equilibrium Synthesis and Modulation of Condensed Matter, Key Laboratory for Advanced Materials and Mesoscopic Physics of Shaanxi Province, School of Physics, Xi’an Jiaotong University, Xi’an 710049, P. R. China

^#^Xiai Zhang and Zhongshuang Xucontributed equally to this work

*Corresponding author. E-mail: [kongcc@xjtu.edu.cn](mailto:kongcc@xjtu.edu.cn) (Chuncai Kong)

**S1 Experimental Section**

S1.1 Chemicals

All chemicals used in this study were commercially purchased and used without further purification. Iron(Ⅲ) chloride (FeCl_3_, AR, 99%), Nickel(Ⅱ) chloride(NiCl_2_, 99%) and Potassium hydroxide (KOH, 95%) were purchased from Shanghai Macklin Biochemical Co., Ltd. Urea (H_2_NCONH_2_, AR, 99%) and Chitosan (C_6_H_11_NO_4_)n, ≥95% were purchased from Shanghai Aladdin Biochemical Technology Co., Ltd. Boric acid (H_3_BO_3_, GR, ≥99.8%), Methanol (CH_4_O, AR, ≥99.5%) and Ethanol (C_2_H_5_OH, AR) were obtained from Tianjin Fuyu Fine Chemical Co., Ltd. Potassium hydrogenperoxomonosulphate (H_3_K_5_O_18_S_4_, 98%) was purchased from Shanghai Yi En Chemical Technology Co., LTD., P-Benzoquinone (C_6_H_4_O_2_, 99%) and Furfuryl alcohol (C_5_H_6_O_2_, AR, 98%) were obtained from Shanghai Aladdin Biochemical Technology Co., Ltd. Deuterium oxide (D_2_O, 99.9 atom % D) and nafion solution (5 wt%) were purchased from Shanghai Aladdin Biochemical Technology Co., Ltd. All experiments were carried out using ultrapure water.

### S1.2 Material Synthesis

Synthesis of NiFe-BNC. 0.4 g of chitosan and 30 mg of urea were dissolved in 50 mL of ultrapure water, then 0.4 g of nickel chloride was added. The solution was stirred continuously until green solution was formed. 1.43 g of anhydrous ferric chloride was dissolved in 10 mL of water to form brown solution. The two solutions were then mixed and stirred at room temperature for 12 h. The mixture was then evaporated at 85°C to remove the water, followed by continuous drying in a forced air oven. The dried sample was placed in a tubular furnace and heated under an argon atmosphere at 750°C for 4 h, then allowed to cool to room temperature. After washing with 6 M HNO_3_ and soaking, the sample was vacuum dried. The dried powder (0.15 g) was mixed with a certain amount of boric acid (0 g, 0.015 g, 0.025 g) and ground for 5 min. The mixture was then heated under an argon atmosphere at 900°C for 2 h to obtain the NiFe-BNC. NiFe-BNC catalysts with different metal loading amounts were also prepared as control samples (where the molar amounts of Fe/Ni were 3/1 mmol and 6/2 mmol). In addition, the preparation of NiFe-NC was similar to that of NiFe-BNC, except for the addition of the boric acid. The preparation of Ni-NC was similar to that of NiFe-NC, except for the addition of the Fe salt. The preparation of Fe-NC was similar to that of NiFe-NC, except for the addition of the Ni salt.

### S1.3 Material Characterization

Various material characterization techniques were employed to examine the morphology, crystal structure, elements, valence states, and reaction intermediate processes of the prepared samples and reduced catalysts. We conducted Synchrotron X-ray characterization utilizing the Table-XAFS-500 equipment manufactured by Specreation Instrument Co., Ltd. Scanning electron microscopy (SEM) on a ThermoFisher Apreo S, STEM, and high-resolution TEM (HR-TEM, JEOL JEM-F200) were used to investigate the morphology and crystal structure. An aberration-corrected high-angle annular dark-field scanning transmission electron microscopy (AC-HAADF-STEM, JEM-ARM300F2) provided further insights. X-ray photoelectron spectroscopy (XPS) measurements were conducted using a Thermo Fisher Scientific ESCALAB Xi^+^ spectrometer with monochromatic Al Kα radiation. X-ray diffraction (XRD, Bruker-D8 ADVANCE) analysis was carried out, operating at 40 kV voltage and 15 mA current with Cu Kα radiation. The surface functional groups of the prepared series of samples were performed using an FTIR spectrometer (Bruker VERTEX 70). Raman tests were performed using the excitation wavelength of 532 nm ((Bruker Senterra). The contact angles were measured with a contact angle measuring device (DSA100S). The electrochemical workstation (CHI 760E) was used to test the performance of electrocatalytic CO_2_ reduction.

## S1.4 VOC Catalytic Oxidation Test

The degradation of gaseous VOCs was conducted in a continuous flow system. The reaction apparatus mainly consists of gas supply system, wet scrubber, and an exhaust gas detection system. After entering the wet scrubber, the gaseous VOCs were converted into microbubbles, which then fully contact the solution to undergo a catalytic reaction. Magnetic stirring was applied during the reaction to ensure the uniform dispersion of the catalyst. The reaction was conducted at a constant temperature of 30°C.

The removal efficiency of VOCs was calculated according to the following equation:

Where the [Styrene]inlet and [Styrene]outlet (ppmv) represent the inlet and outlet concentrations of styrene, respectively.

Electron paramagnetic resonance (EPR) testing was applied to testify to the existence of ROS with 5,5-dimethyl-1-pyrroline-N-oxide (DMPO) and 2,2,6,6-tetramethyl-4-piperidone hydrochloride (TEMP) as the spin trapping reagents. DMPO was used as the spin trapping agent for HO•, SO_4_^•–^, and O_2_^•–^ radicals, while TEMP for ^1^O_2_. More experimental details about the reagents, catalyst characterizations, VOC degradation procedure, electrochemical test, intermediate detection, and health risk assessment method can be found in the Supporting Information.

### S1.5 Electrocatalytic CO_2_ Reduction

**Electrode Preparation.** The prepared catalysts (20 mg) were weighed. Then, 120 µL of Nafion solution, 750 μL of water and 750 μL of ethanol were added. The solution was ultrasonicated for 10 minutes to create a homogeneous ink. This ink was evenly sprayed onto carbon paper, with dimensions of 1.5 × 1.5 cm^2^ for the flow cell electrolyzer and 1.3 × 1.3 cm^2^ for the membrane electrode assembly electrolyzer (MEA) electrolyzer, using a spray gun. A real 1.0 × 1.0 cm^2^ gas diffusion electrode (GDE) was assembled into a flow cell electrolyzer or MEA electrolyzer as the working electrode.

**eCO_2_RR tests.** All electrochemical measurements were conducted under ambient temperature and pressure conditions. eCO_2_RR tests were carried out in a flow cell electrolyzer using a CHI electrochemical workstation (1440 series). Each current density was maintained at a constant level for 1000 seconds.

The prepared GDE served as the cathode in the flow cell electrolyzer, while the Ti mesh coated with IrO_2_ was used as the anode. For eCO_2_RR testing, the cathode electrolyte and anode electrolyte contained a solution with 1 mol/L KOH. Peristaltic pumps were used to circulate both electrolytes through the cathode and anode chambers. The cathodic chamber and anodic chamber were separated by the anion exchange membrane (Fumapem FAA-3-PK-130). The flow rate of the CO_2_ gas was controlled using a mass flow meter controller and corrected with a soap bubble flow meter at a rate of 100 sccm. The reference electrode employed was Hg/HgO. Voltage values were converted using the following equations:

Where
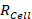
 represents the solution resistance of the electrolyte, which is determined through electrochemical impedance spectroscopy testing, and
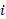
 represents the current at the respective voltage.

In the MEA electrolyzer, the anode chamber was supplied with 1 mol/L KOH, using a peristaltic pump. Simultaneously, the cathode chamber in the MEA electrolyzer received a continuous inflow of humidified CO_2_ gas. The flow rate of the CO_2_ gas was controlled using a mass flow meter controller and corrected with a soap bubble flow meter, at a rate of 50 sccm. The prepared GDE served as the cathode in the MEA electrolyzer, while the IrO_2_ was used as the anode. The anode utilized an anion exchange membrane.

During the evaluation of the catalyst’s eCO_2_RR performance, we quantified and collected the electrolyte from both the cathode and anode, while separating the gas product from the liquid product by passing it into ultrapure water. To ensure conductivity and maintain ionic concentration, we periodically replaced the electrolyte with fresh electrolyte during stability tests. The gas products of the reaction were dried using a desiccant, collected in a gas bag, and then analyzed both qualitatively and quantitatively using gas chromatography (Tianmei 7980) equipped with TCD and FID detectors. We calculated the respective Faraday efficiencies (FE) for gaseous product obtained using the following equations:

where
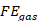
 represents the Faradaic efficiency for the gas product;
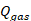
 represents the charge transferred for the formation of the product gas;
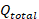
 represents the total charge passed through the working electrode;
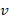
 represents the outlet gas flow rate;
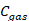
 represents the concentration of the product as detected by GC;
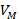
 represents the molar volume of the gas at room temperature, which is 24.5 L/mol;
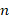
 represents the number of electrons transferred for reduction to a molecule product;
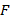
 represents the Faradaic constant, which is 96485 C/mol and
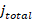
 represents the total current density during CO_2_ electrolysis.

In order to analyze whether there are liquid phase products in the reaction process, 500 µL was withdrawn using a pipette. Then, 1 µL of DMSO and 200 µL of D_2_O were added to the withdrawn liquid. The resulting mixture was qualitatively and quantitatively analyzed using a ^1^H NMR (Bruker Advance III 400 HD spectrometer).

### S1.6 *In-situ* Spectroscopy

***In-situ* Raman spectroscopy (eCO_2_RR).** Raman spectra were acquired using a Raman flow cell with 532 nm excitation laser. During the in-situ experiment, a solution contained with 1 mol/L KOH was continuously circulated through the cathodic chamber, while a 1 mol/L KOH solution was circulated through the anodic chamber. This circulation was achieved using peristaltic pumps at a rate of 5 mL/min. The flow rate of CO_2_ was maintained at 100 sccm using a mass flow controller.

***In-situ* Raman spectroscopy (PMS activation).** Quantitative catalyst powder and PMS were mixed with 5 mL ultra-pure water, and the mixture was put into a 10 mL centrifuge tube, which was shock for 1 min to complete the reaction, and a small amount of turbidity was absorbed with a capillary tube. Under the irradiation of 532 nm laser, a resolution of 1 cm^-1^ was used to scan for 1 min in the range of 400 - 4000 cm^-1^. Similar to the test method for control samples, the PMS is dissolved in pure water for scanning.

### S1.7 Theoretical Calculations.

This study employed density functional theory (DFT) calculations using the Dmol^3^ module in Materials Studio software for first-principles simulations. The exchange-correlation interactions were described by the Perdew-Burke-Ernzerhof (PBE) functional within the generalized gradient approximation (GGA) framework. Double numerical plus polarization (DNP) basis sets and density-functional semi-core pseudopotentials (DSPP) were adopted for calculations, with van der Waals (vdW) corrections incorporated. Gibbs free energy corrections were implemented for the CO_2_ reduction process. The Brillouin zone integration utilized a Monkhorst-Pack k-point grid of 2×2×1, with convergence thresholds set as follows: energy tolerance (2.0×10^-5^ Ha), maximum force (0.004 Ha/Å), maximum displacement (0.005 Å), and self-consistent field (SCF) convergence criterion (1.0×10^-5^). These computational parameters ensured rigorous electronic structure optimization and thermodynamic evaluations.

**S2 Supplementary Figures**

**
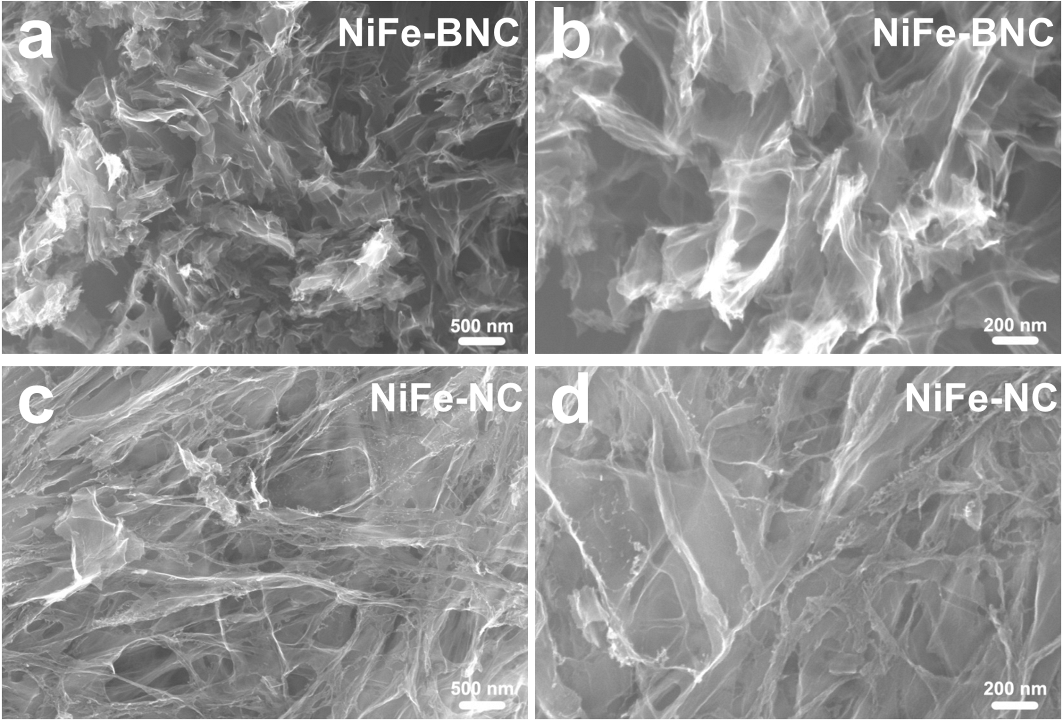
**

#### Fig. S1 SEM images showing the morphology of the NiFe-BNC (a-b) and NiFe-NC (c-d). SEM images show the layered loose porous carbon structure

**
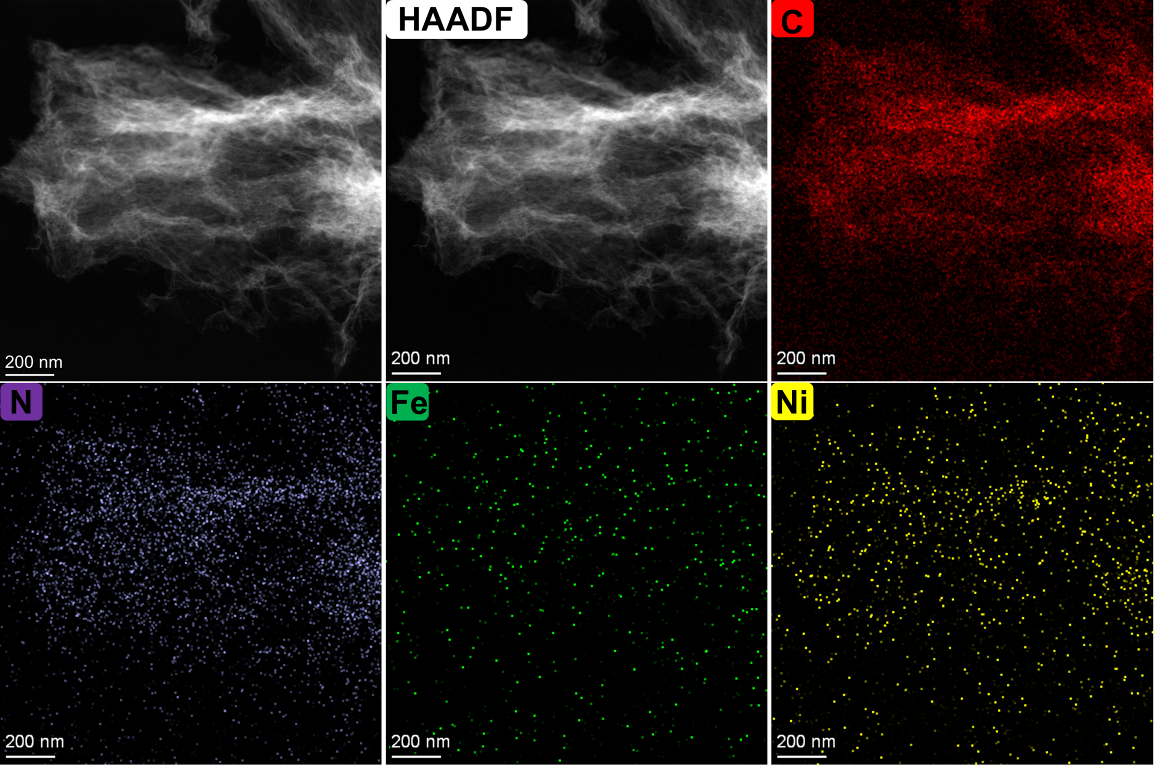
**

#### Fig. S2 EDS mapping of NiFe-NC


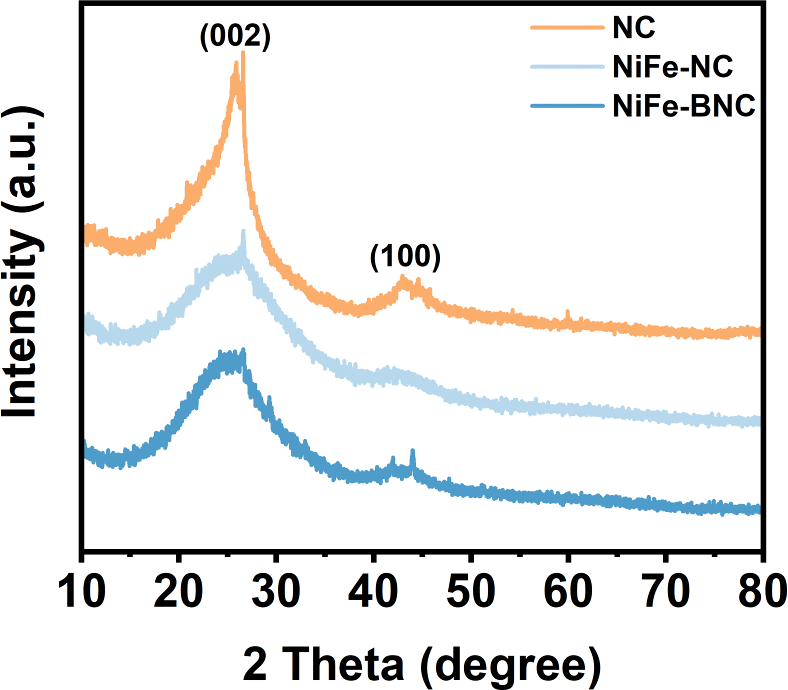


#### Fig. S3 XRD pattern of NC, NiFe-NC and NiFe-BNC


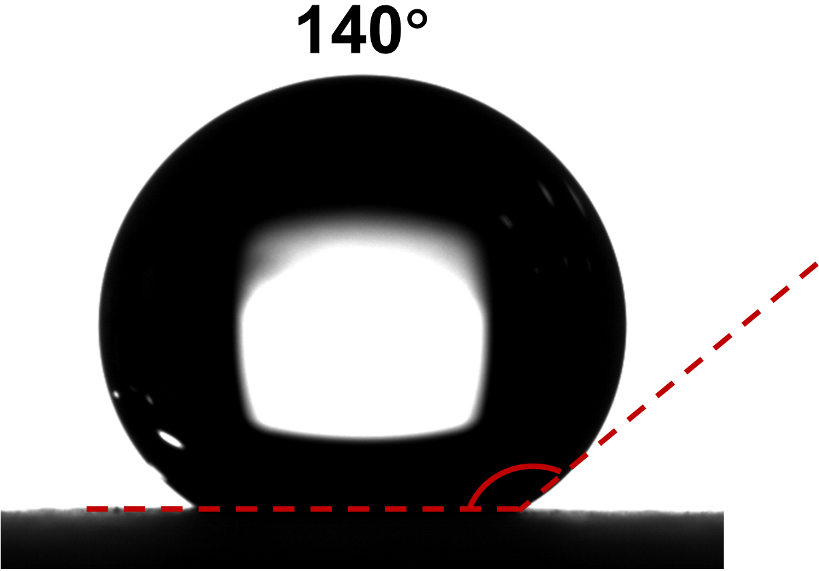


#### Fig. S4 Contact angle measurement of NiFe-BNC, showing a water contact angle of 140°, indicating hydrophobicity

####
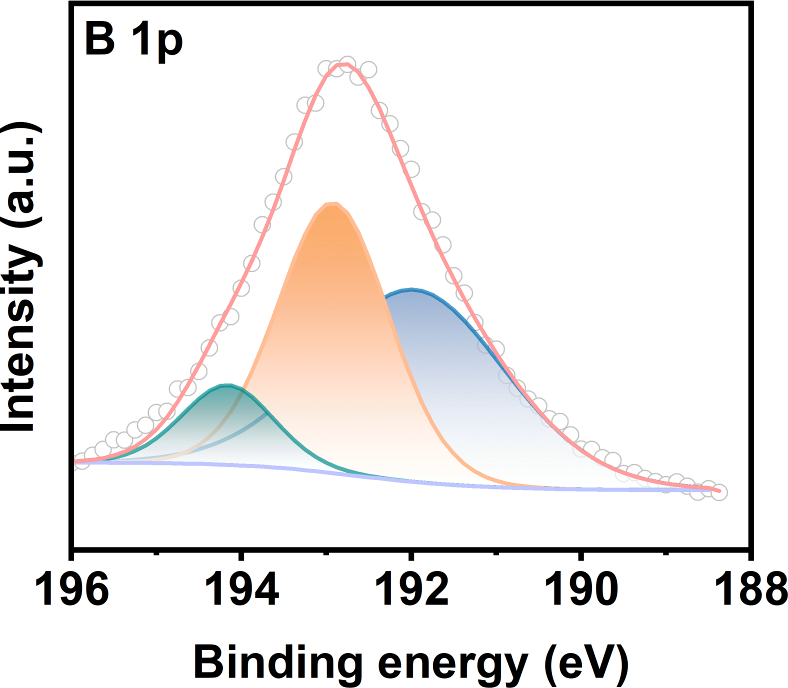


#### Fig. S5 B 1p XPS spectrum of NiFe-BNC


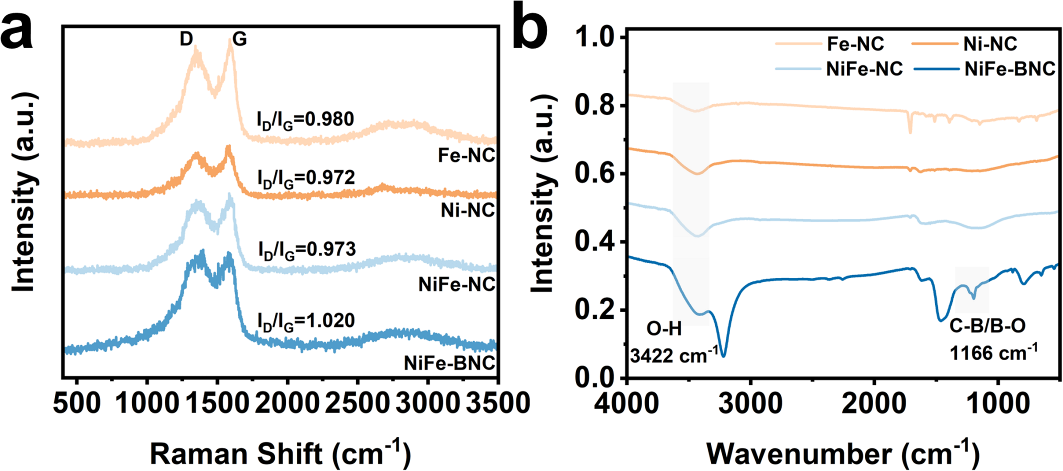


#### Fig. S6 Raman spectra and FTIR spectra of different catalysts


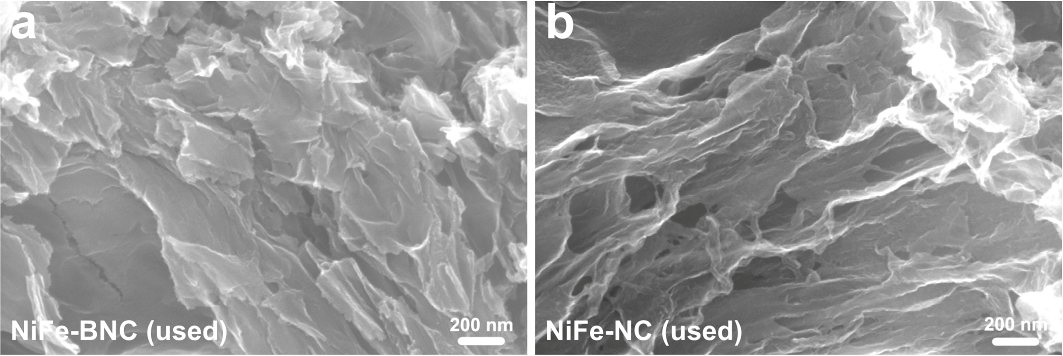


#### Fig. S7 Morphology of NiFe-BNC catalyst after catalytic degradation experiment


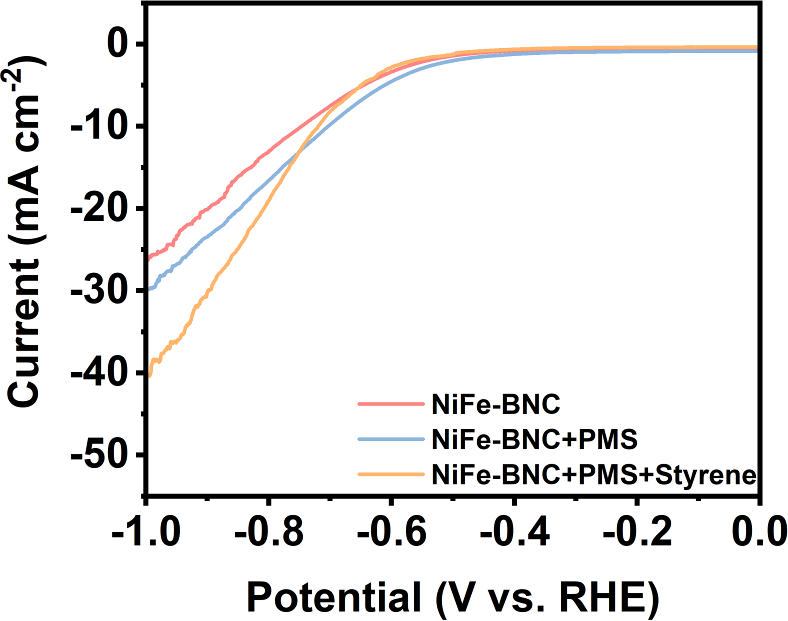


#### Fig. S8 LSV Curves of NiFe-BNC in Different Reaction Systems


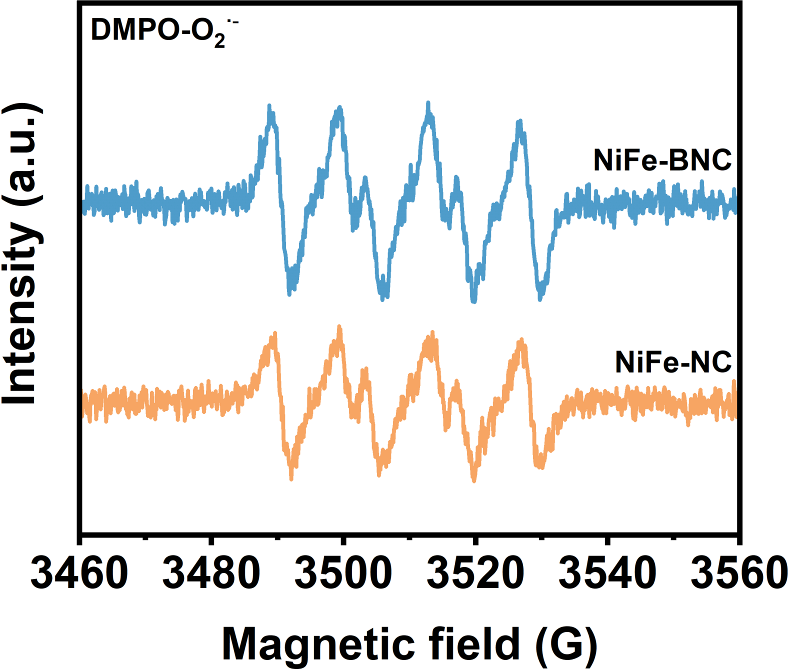


#### Fig. S9 EPR spectra in the catalyst/PMS system


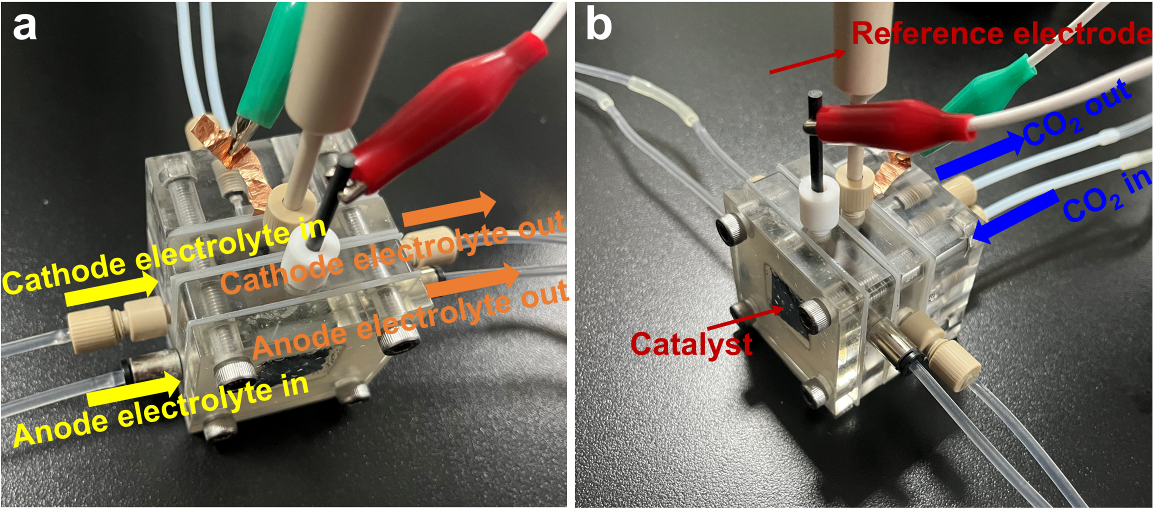


#### Fig. S10 The actual setup of the flow cell electrolyzer used for CO_2_ electroreduction. The electrolyzer consists of a gas diffusion electrode (GDE) for CO_2_ introduction, a cathode and anode electrolyte compartment separated by a membrane, and a reference electrode. The cathode and anode compartments allow for the inflow and outflow of electrolytes, facilitating the electrochemical reduction process


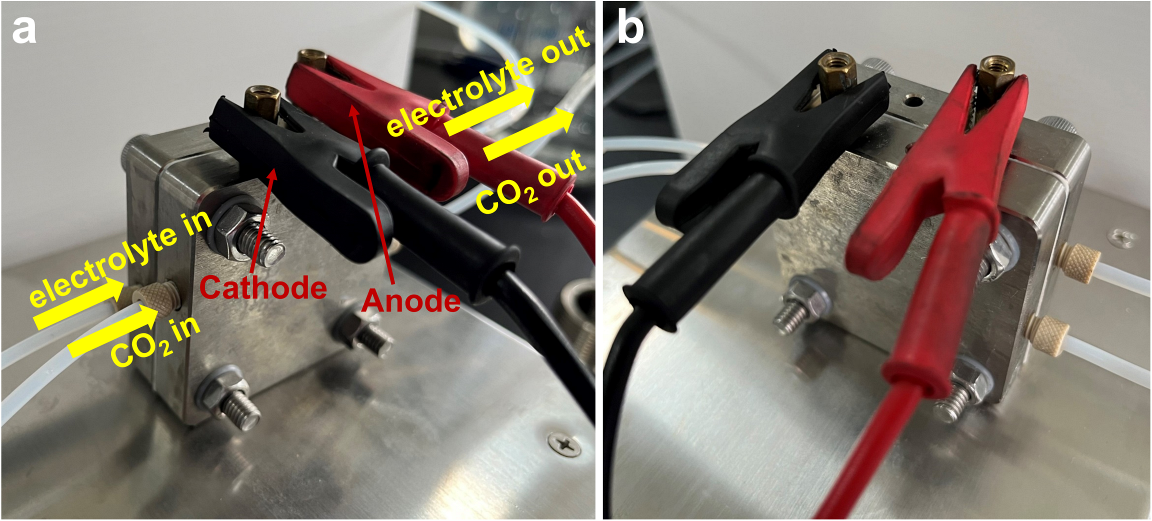


#### Fig. S11 The actual photograph of the membrane electrode assembly (MEA) electrolyzer setup. The diagram on the left shows the components of the MEA electrolyzer, including the anode with IrO₂ catalyst, the anion exchange membrane (AEM), and the gas diffusion electrode (GDE) at the cathode. The system facilitates the flow of electrolyte and CO_2_ through the anode and cathode, respectively


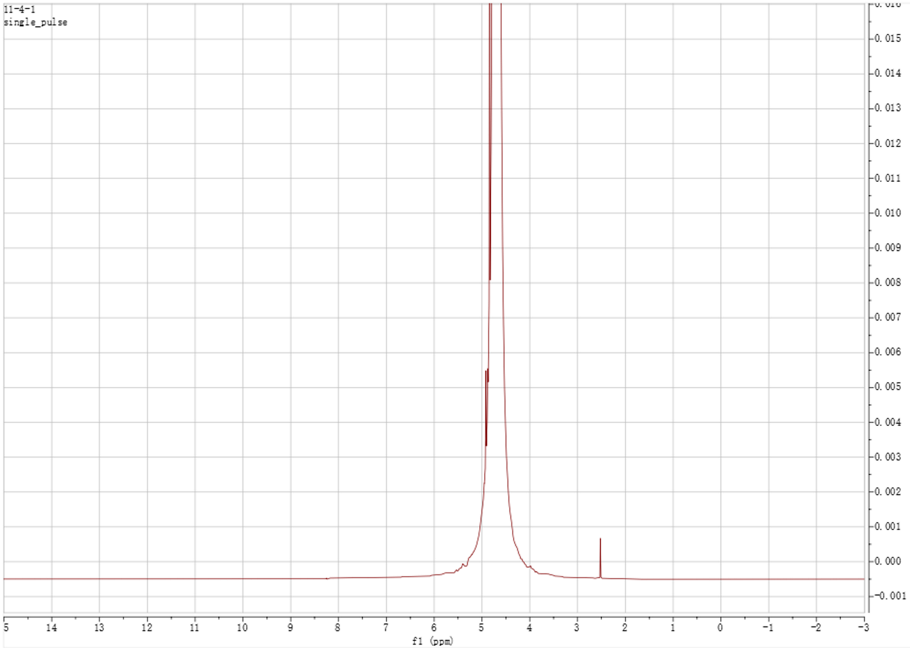


#### Fig. S12 ^1^H NMR spectra of the different products obtained after the eCO_2_RR


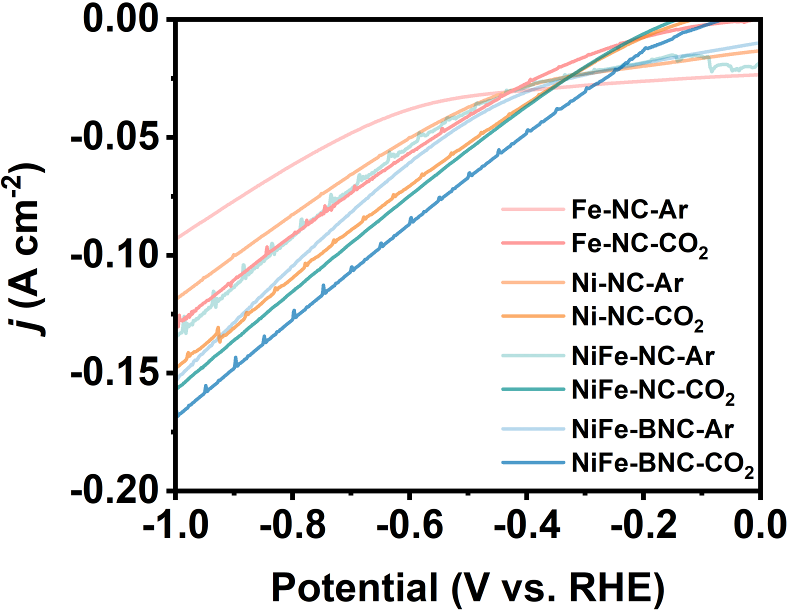


#### Fig. S13 LSV curves of different catalysts: Ar-saturated electrolyte (flow cell), CO_2_-saturated electrolyte (flow cell)


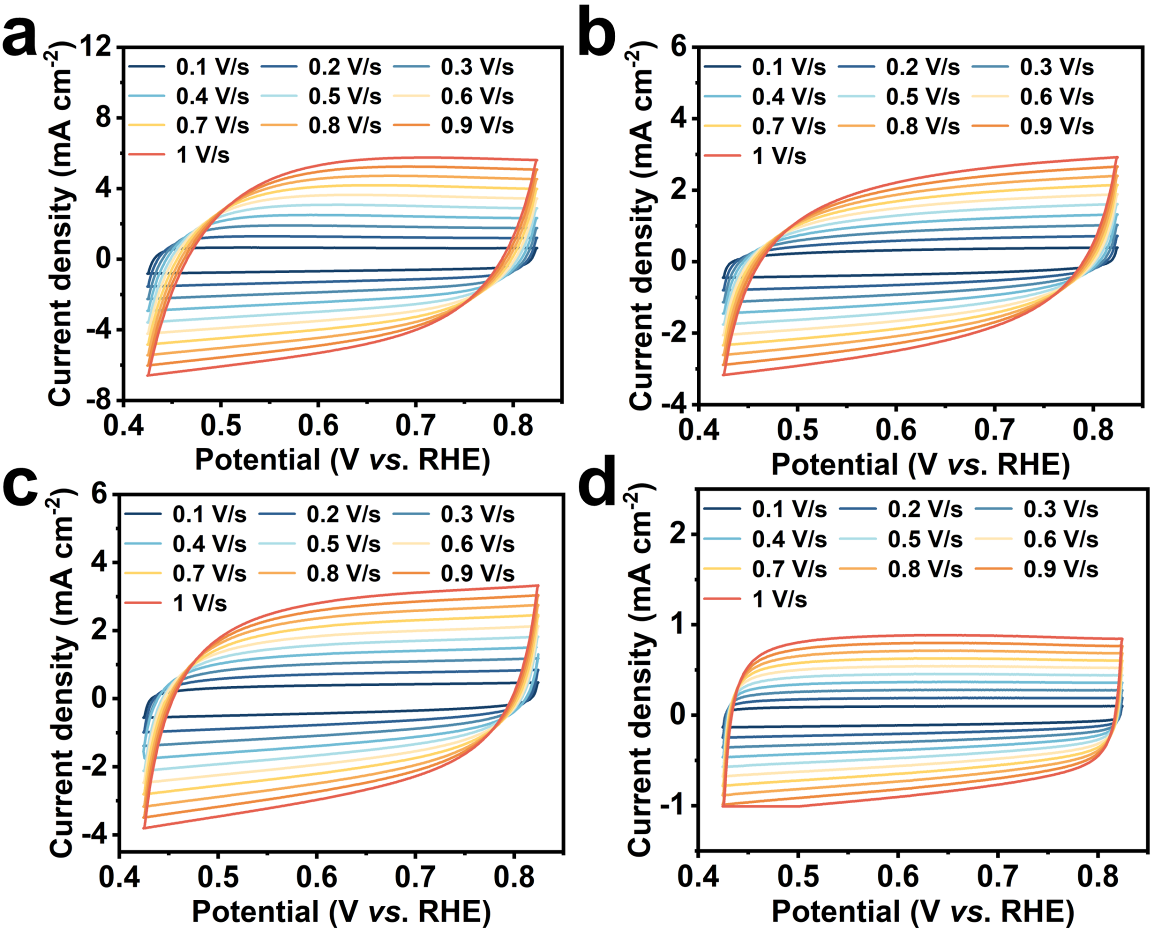


Fig. S14 Cyclic voltammetry curves. (a) NiFe-BNC; (b) NiFe-NC; (c) Ni-NC and (d) Fe-NC with the scan rates from 0.1 V/s to 1 V/s


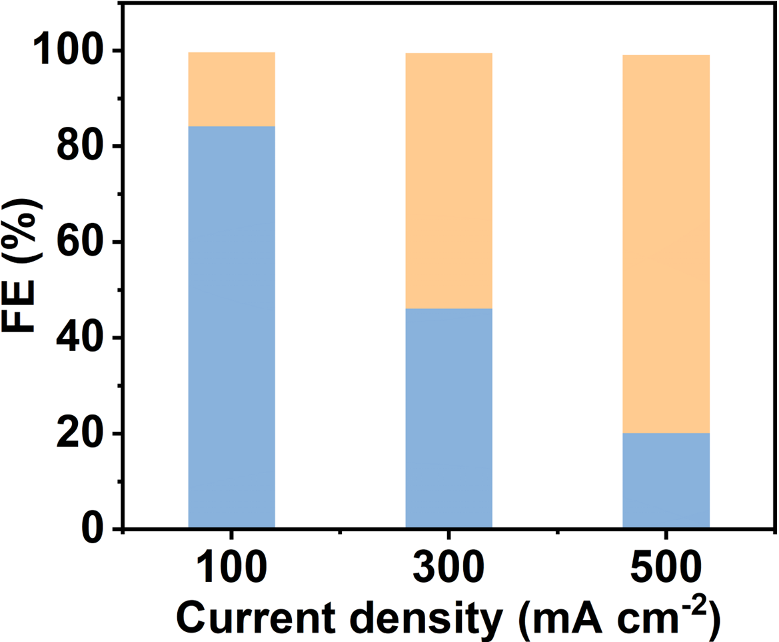


#### Fig. S15 FE_CO_ of Fe-NC and Ni-NC physical mixed catalysts


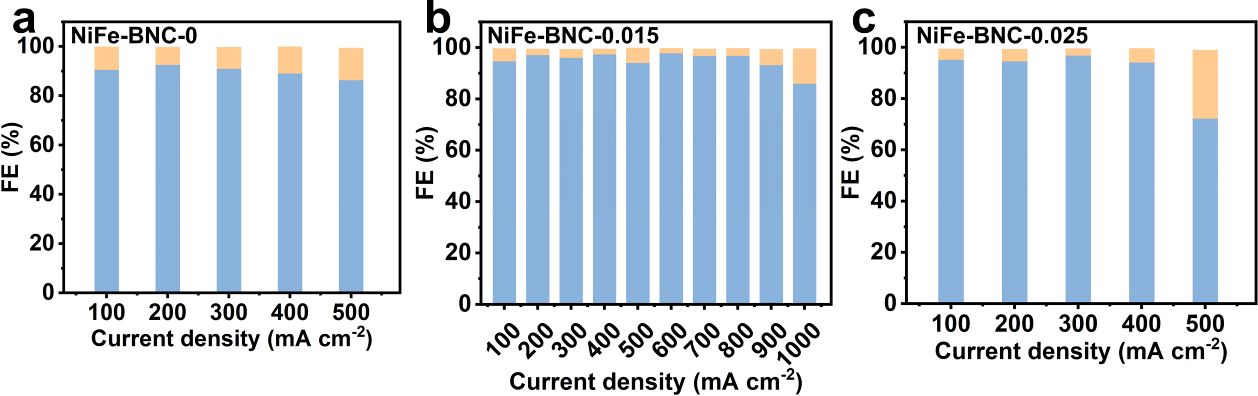


#### Fig. S16 The eCO_2_RR performance of NiFe-BNC with varying boron doping levels


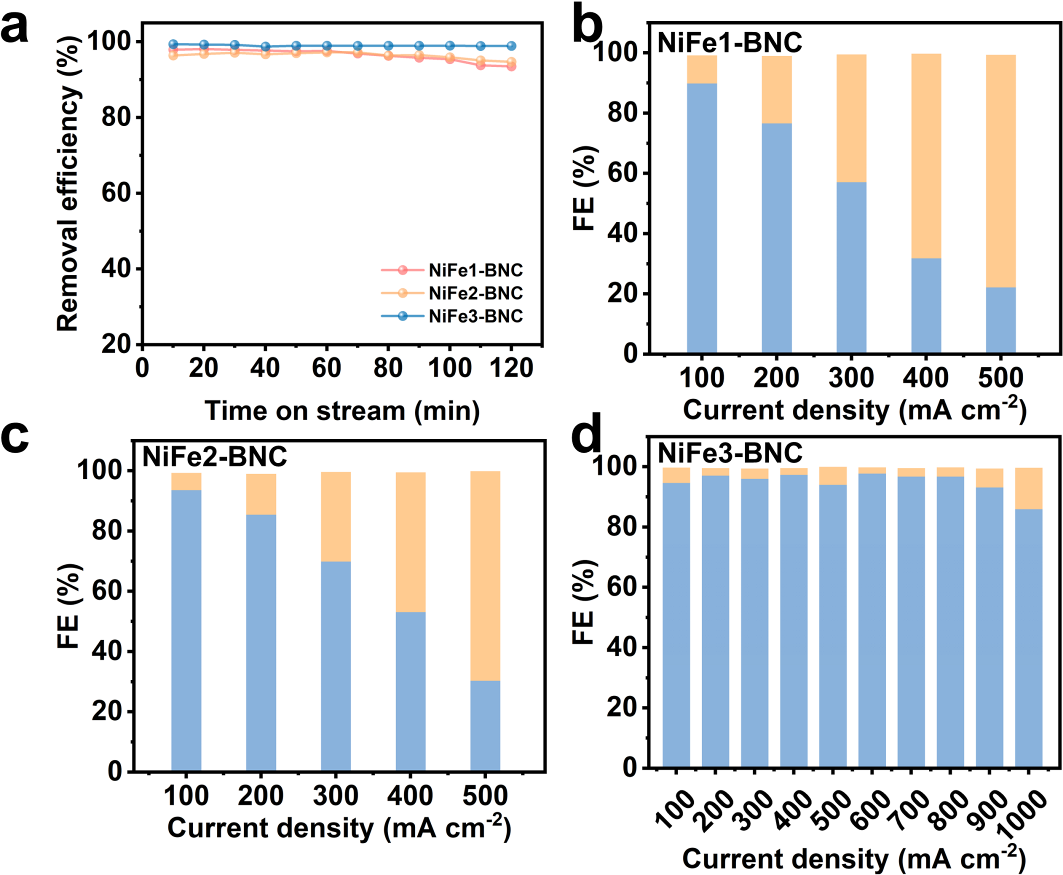


#### Fig. S17 (a) The styrene degradation performance and (b-d) the corresponding Faradaic efficiencies of CO and H_2_ at different current densities of NiFe-BNC catalysts with varying metal contents


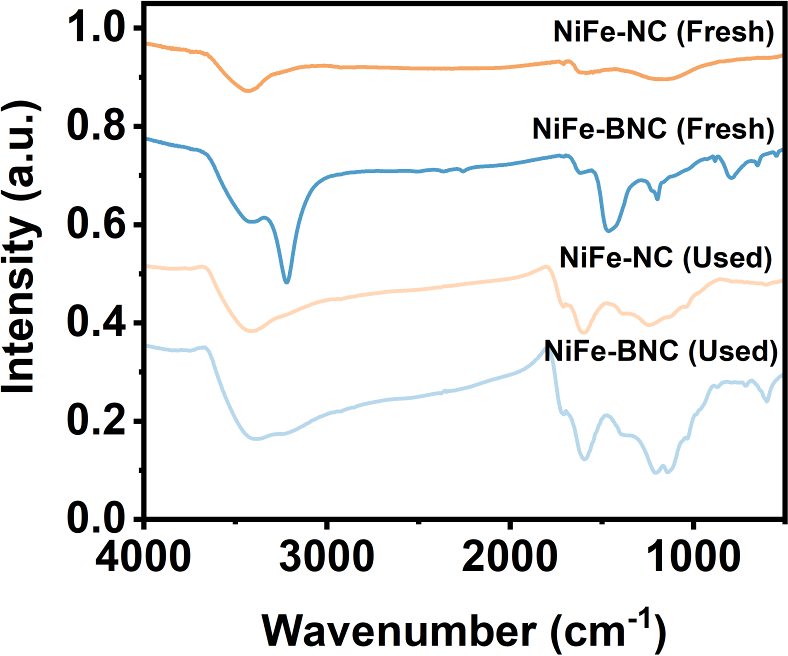


#### Fig. S18 Infrared spectra of NiFe-NC and NiFe-BNC catalysts before and after catalytic degradation reaction


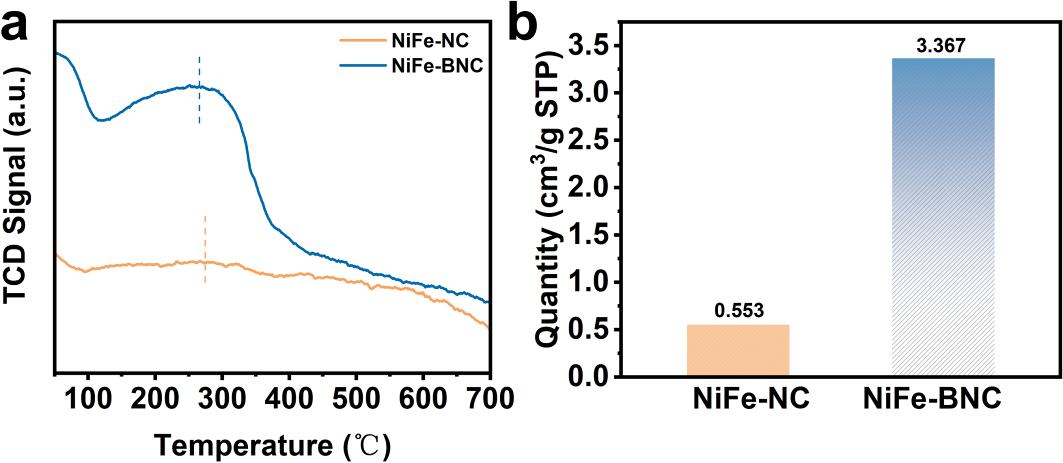


#### Fig. S19 (a) The CO_2_-TPD profiles and (b) corresponding CO_2_ adsorption capacities of the NiFe-NC and NiFe-BNC catalysts


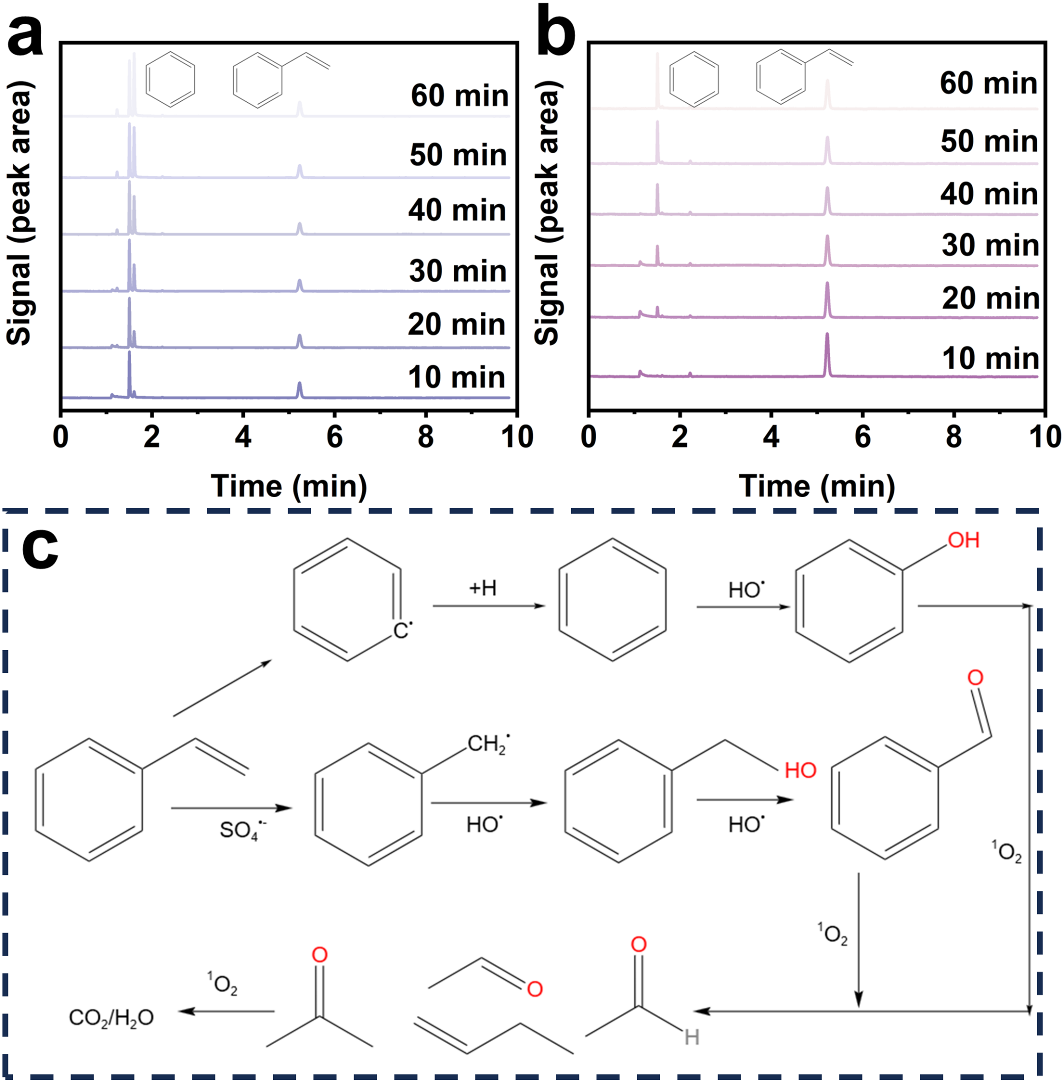


#### Fig. S20 (a-b) Actual gas chromatogram of the catalytic reaction with the addition of ethanol and methanol quencher; (c) The possible degradation pathways of styrene


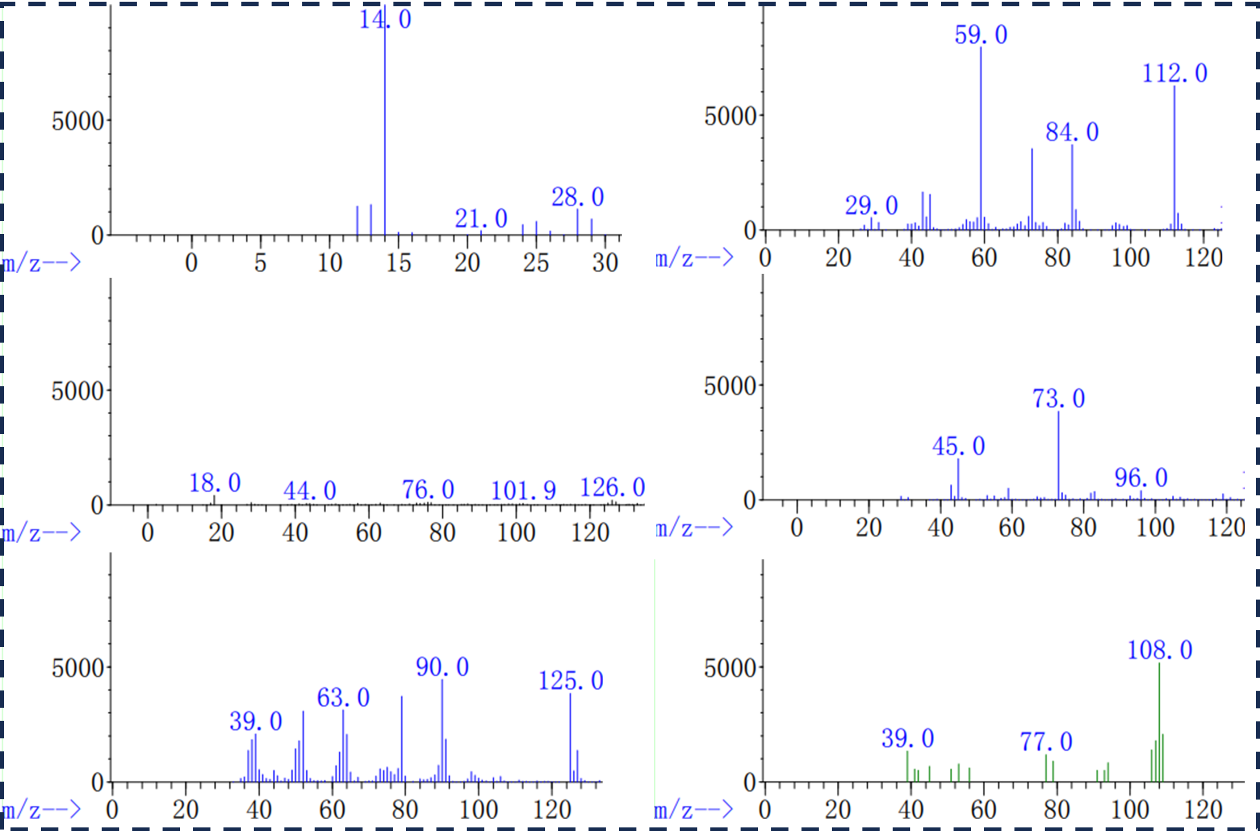


#### Fig. S21: The spectrum of gas chromatography-mass spectrometry (GC-MS)


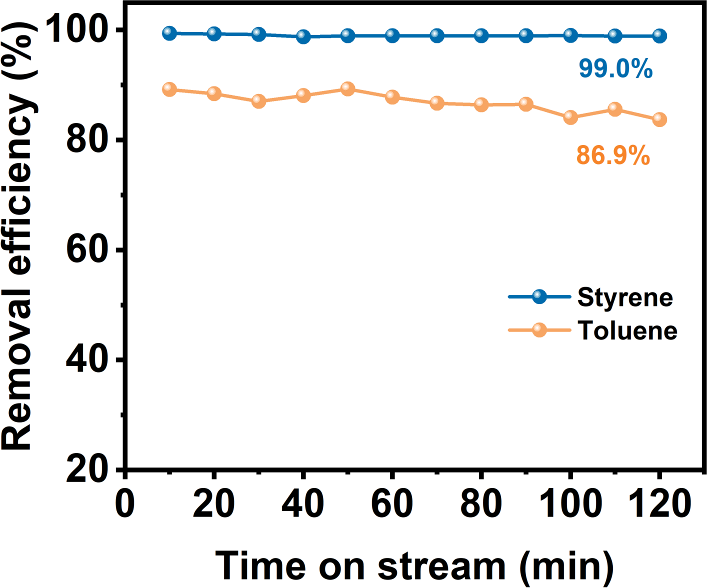


#### Fig. S22 Removal efficiency of styrene and toluene by NiFe-BNC/PMS systems


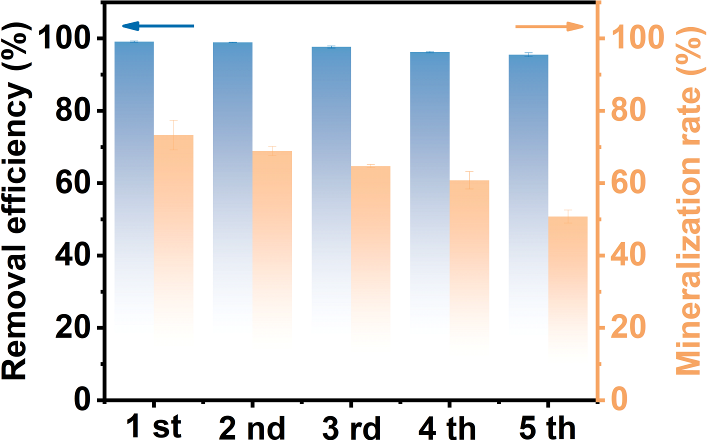


#### Fig. S23 Cyclic degradation experiment of the catalysts


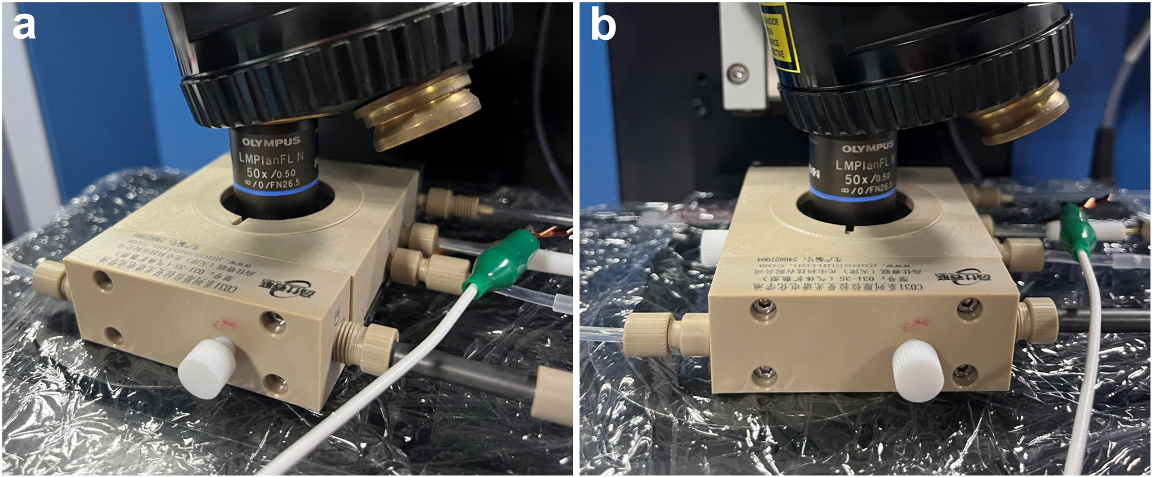


#### Fig. S24 Schematic diagram of the *in-situ* Raman spectroscopy setup for CO_2_ reduction reaction studies, with a gas diffusion electrode (GDE) and a membrane separating the cathode and anode compartments


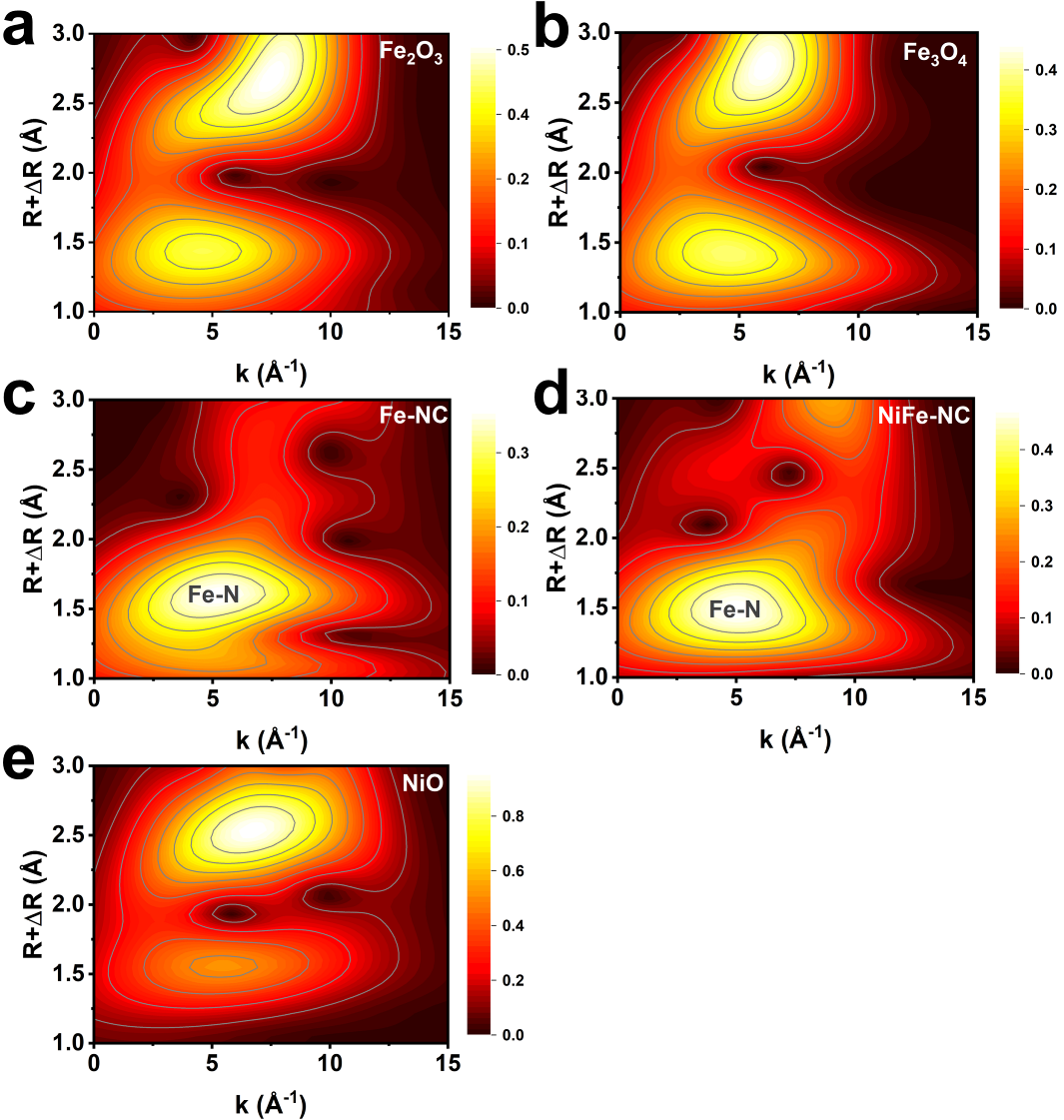


#### Fig. S25 WT-EXAFS plots of (a) Fe_2_O_3_, (b) Fe_3_O_4_; (c) Fe-NC; (d) NiFe-NC; (e) NiO


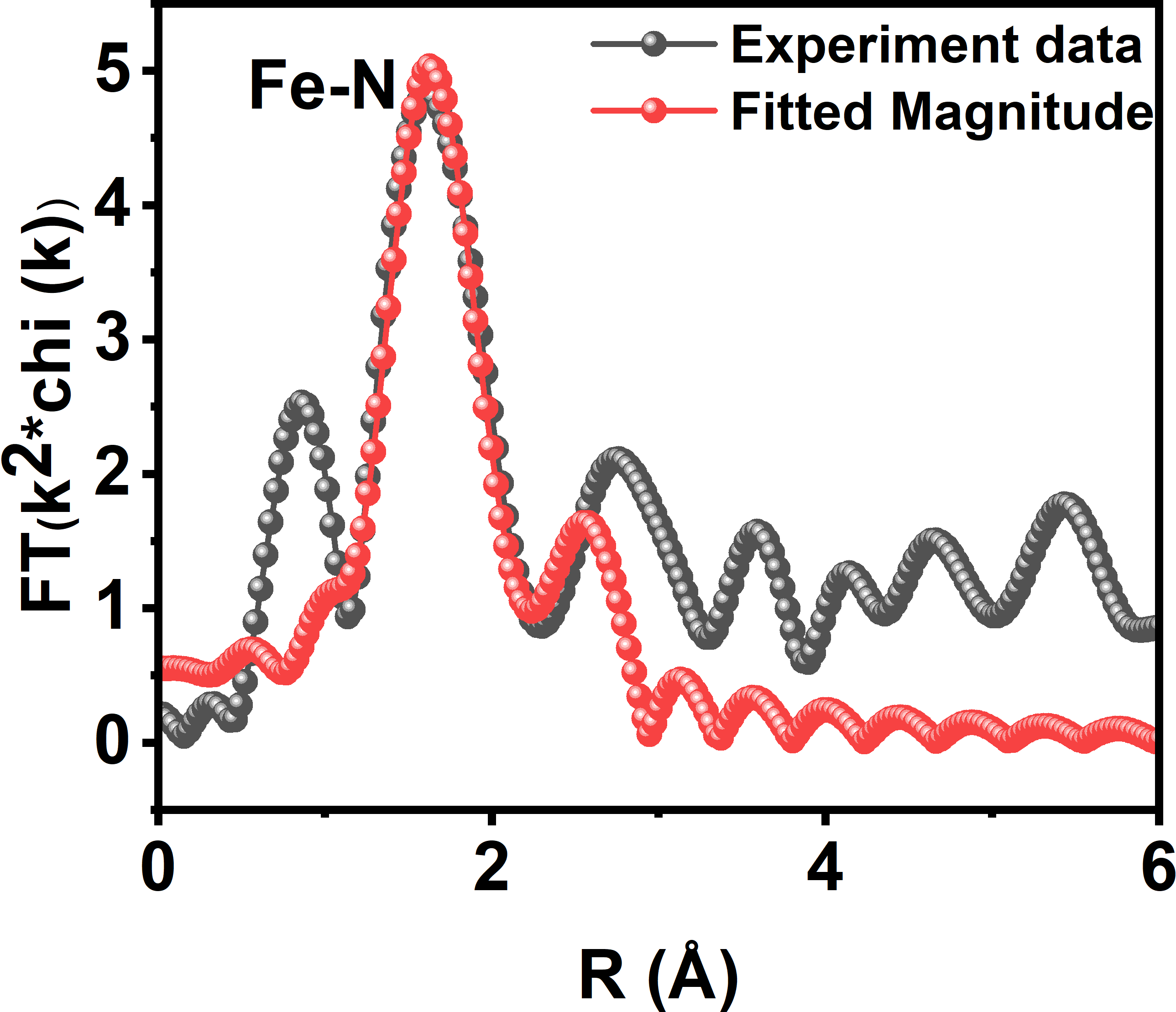


#### Fig. S26 Experimental and fitting EXAFS curves of Fe-NC in R space (Fe-N_4_)


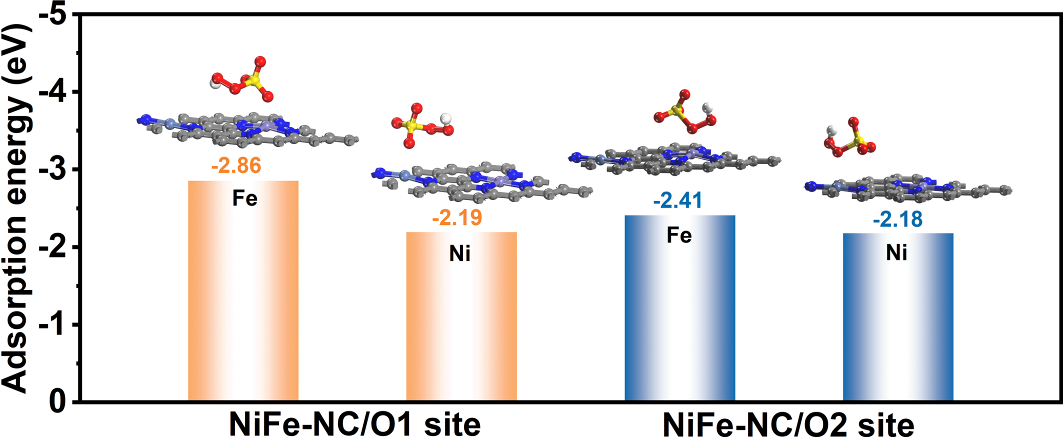


#### Fig. S27 Adsorption energy of different oxygen sites of PMS at Fe/Ni metal sites of NiFe-NC catalyst


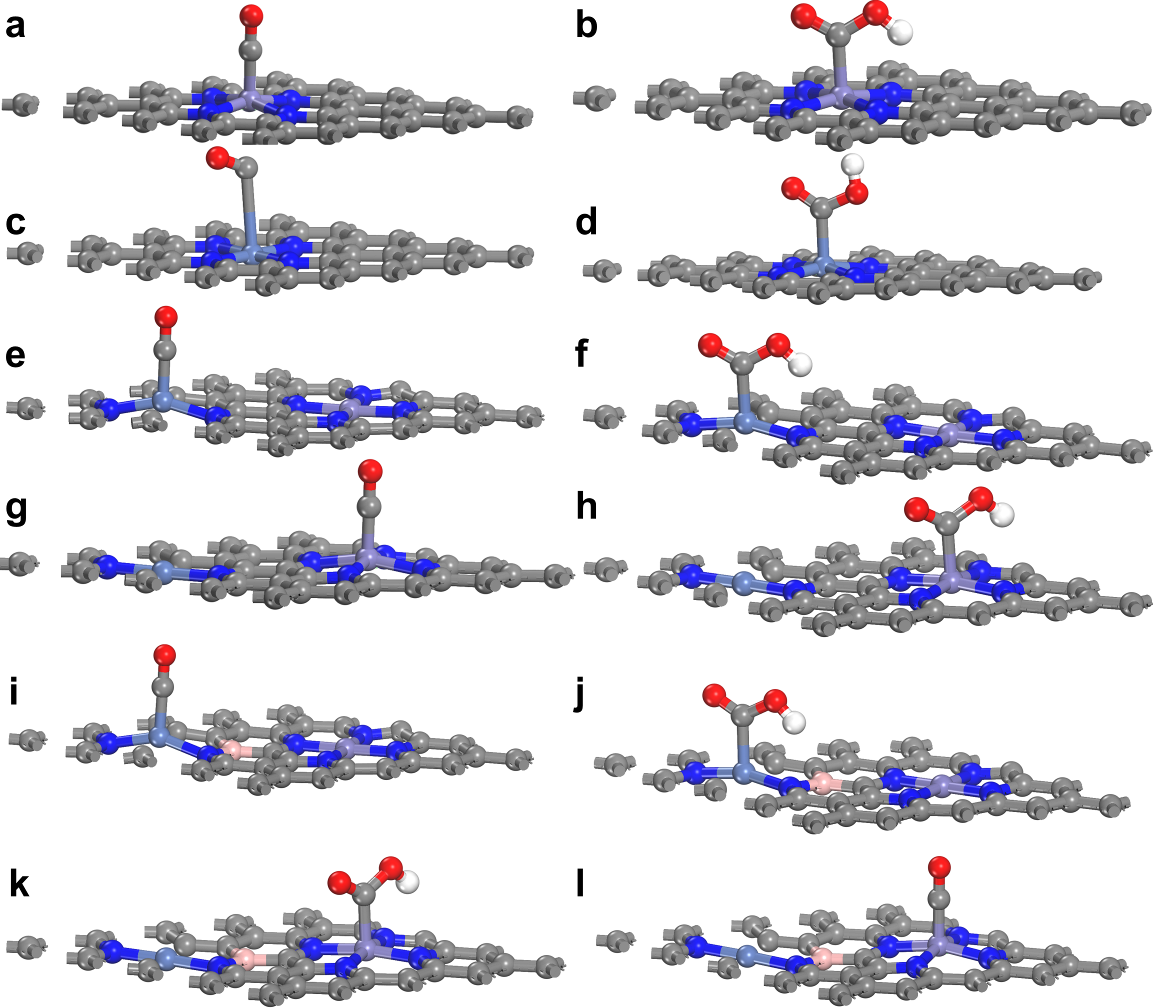


#### Fig. S28 Gibbs free energy calculation model diagram


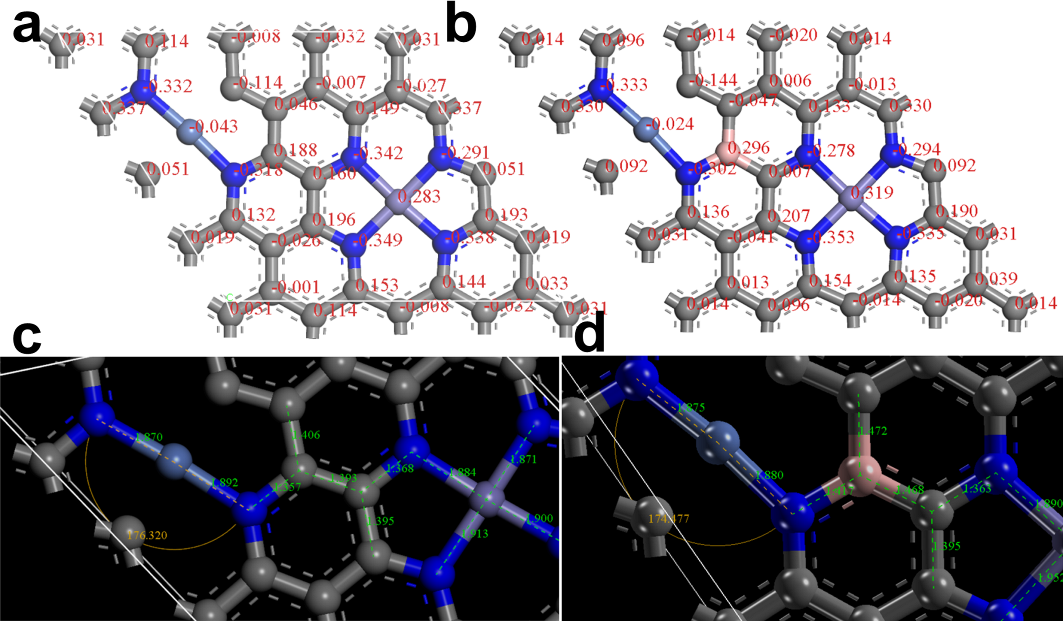


#### Fig. S29 (a-b) Mulliken charge diagram and (c-d) the optimized structural model diagrams of NiFe-NC and NiFe-BNC catalysts


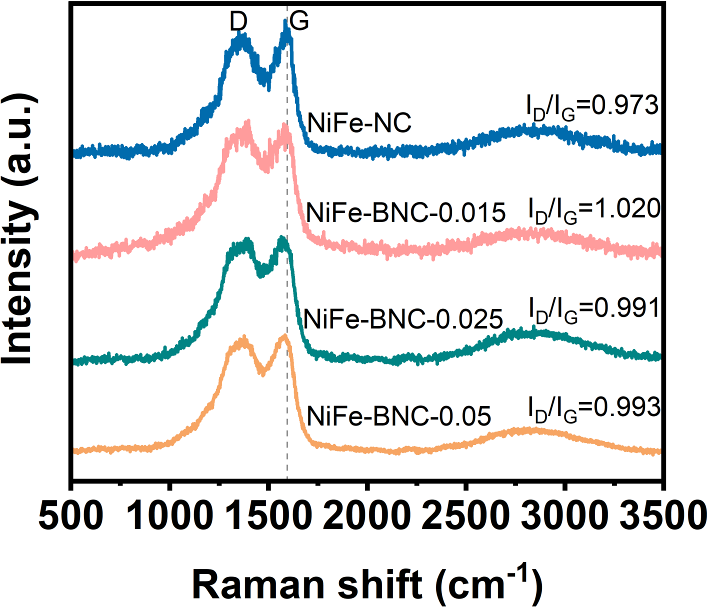


#### Fig. S30 Raman spectra of NiFe-BNC catalysts with different boron doping contents

**Table S1 Structural parameters extracted from the Fe/Ni K-edge EXAFS fitting**

| Sample | Scattering path | Distance (Å) | | C.N. | | (Å2) | ∆E_0_ (Ev) | R-factor |
| --- | --- | --- | --- | --- | --- | --- | --- | --- |
| Fe foil | Fe-Fe | 2.46 |  | | 8 | 0.004 | 6.7 | 0.004 |
|  | Fe-Fe | 2.85 |  | | 6 | 0.004 | 6.7 |  |
| NiFe-BNC (Fe) | Fe-N | 1.99 |  | | 3.6 | 0.001 | 13.2 | 0.017 |
| Ni foil | Ni-Ni | 2.49 |  | | 12 | 0.006 | 7.1 | 0.002 |
| NiFe-BNC (Ni) | Ni-Ni | 2.49 |  | | 3.1 | 0.005 | -8.1 | 0.007  0.007 |
|  | Ni-N | 1.90 |  | | 2.4 | 0.005 | -8.1 |  |

**Table S2 Comparison of the removal effect of VOCs by liquid-phase AOPs**

| Catalytic system | Pollutant | Degradation efficiency（%） | Optimal mineralization rate（%） | Pollutant concentration（ppm） | Refs. |
| --- | --- | --- | --- | --- | --- |
| Co_3_O_4_/activated carbon/PMS | Toluene | 90 | 57 | 30 | [S1] |
| CFS/PMS | Toluene | 95 | 47 | 30 | [S2] |
| Fe^3+^/MoS_2_/PMS  UV/PMS  Carbon nanotubes/PMS | Styrene  Ethyl acetate Toluene  Styrene | 97  96  95  98 | 36  62  88  76 | 30  30  30  30 | [S3]  [S4]  [S5] |
| Co@NCNT/PMS | Chlorobenzene | 90 | 38 | 30 | [S6] |
| MnCo_2_O_4.5_ | Toluene | 97.3 | 85.3 | 30 | [S7] |
| Fe/ZSM-5/UV/Fenton  UV/H_2_O_2_  UV/PDS  CoS_2_/AC/PMS | Toluene  Toluene  Chlorobenzene Toluene | 85  81.4  90-97  90 | /  72  75  80 | 30  30  30  30 | [S8-S11] |
| NF-BNC/PMS | Styrene | 99 | 94 | 30 | This work |

**Table S3** Comparative analysis of the eCO_2_RR performance of the NiFe-BNC catalyst compared to other reported electrocatalysts

| **Electrocatalyst** | **Electrolyte** | **Potential/V vs. RHE/**  **J_CO_ (mA/cm^2^)** | **FE_CO_  (%)** | **Refs.** |
| --- | --- | --- | --- | --- |
| NF-BNC | Flow Cell | 1000 | 98% | **This work** |
|  | MEA | 400 | 97% |  |
| Cu-S-Ni/SNC | Flow Cell | 400 | 98% | [S12] |
| FeNi–NSC | H-type Cell | 40 | 90% | [S13] |
| NiN_4_B_2_C_x_ | H-type Cell | 67.91 | 95% | [S14] |
| Cu/Ni-NC | Flow Cell | 300 | 95% | [S15] |
| CuNi-DSACs | Flow Cell | 213 | 99% | [S16] |
| Ni-NBr-C | MEA | 350 | 97% | [S17] |
| Ni-Zn bimetal site | H-type Cell | -0.8V | 99% | [S18] |
| Fe-Zn dual-atom sites | Flow Cell | 400 | 94% | [S19] |
| NiFe-DASC | H-type Cell | 50.4 | 94.5% | [S20] |
| CuZn-DAS/NC | Flow Cell | -0.6V | 98.4% | [S21] |

**Supplementary References**

1. R. Xie, J. Ji, H. Huang, D. Lei, R. Fang et al., Heterogeneous activation of peroxymonosulfate over monodispersed Co_3_O_4_/activated carbon for efficient degradation of gaseous toluene. Chem. Eng. J. **341**, 383–391 (2018). <https://doi.org/10.1016/j.cej.2018.02.045>
2. X. Xie, R. Xie, Z. Suo, H. Huang, M. Xing et al., A highly dispersed Co–Fe bimetallic catalyst to activate peroxymonosulfate for VOC degradation in a wet scrubber. Environ. Sci. Nano **8**(10), 2976–2987 (2021). <https://doi.org/10.1039/D1EN00547B>
3. X. Xie, J. Cao, Y. Xiang, R. Xie, Z. Suo et al., Accelerated iron cycle inducing molecular oxygen activation for deep oxidation of aromatic VOCs in MoS_2_ co-catalytic Fe^3+^/PMS system. Appl. Catal. B Environ. **309**, 121235 (2022). <https://doi.org/10.1016/j.apcatb.2022.121235>
4. R. Xie, J. Ji, K. Guo, D. Lei, Q. Fan et al., Wet scrubber coupled with UV/PMS process for efficient removal of gaseous VOCs: Roles of sulfate and hydroxyl radicals. Chem. Eng. J. **356**, 632–640 (2019). <https://doi.org/10.1016/j.cej.2018.09.025>
5. J. Wu, J. Wang, C. Liu, C. Nie, T. Wang et al., Removal of gaseous volatile organic compounds by a multiwalled carbon nanotubes/peroxymonosulfate wet scrubber. Environ. Sci. Technol. **56**(19), 13996–14007 (2022). <https://doi.org/10.1021/acs.est.2c03590>
6. X. Xie, F. Xiao, S. Zhan, M. Zhu, Y. Xiang et al., Deep oxidation of chlorinated VOCs by efficient catalytic peroxide activation over nanoconfined Co@NCNT catalysts. Environ. Sci. Technol. **58**(3), 1625–1635 (2024). <https://doi.org/10.1021/acs.est.3c08329>
7. S. Wang, S. Liu, X. Chen, Y. Guo, X. Xu et al., Mn-Co bimetallic spinel catalyst towards activation of peroxymonosulfate for deep mineralization of toluene: The key roles of SO4•- and O2•- in the ring-opening and mineralization of toluene. Chem. Eng. J. **453**, 139901 (2023). <https://doi.org/10.1016/j.cej.2022.139901>
8. R. Xie, G. Liu, D. Liu, S. Liang, D. Lei et al., Wet scrubber coupled with heterogeneous UV/Fenton for enhanced VOCs oxidation over Fe/ZSM-5 catalyst. Chemosphere **227**, 401–408 (2019). <https://doi.org/10.1016/j.chemosphere.2019.03.160>
9. G. Liu, J. Ji, H. Huang, R. Xie, Q. Feng et al., UV/H_2_O_2_: an efficient aqueous advanced oxidation process for VOCs removal. Chem. Eng. J. **324**, 44–50 (2017). <https://doi.org/10.1016/j.cej.2017.04.105>
10. R. Xie, J. Cao, X. Xie, D. Lei, K. Guo et al., Mechanistic insights into complete oxidation of chlorobenzene to CO_2_ *via* wet scrubber coupled with UV/PDS. Chem. Eng. J. **401**, 126077 (2020). <https://doi.org/10.1016/j.cej.2020.126077>
11. Y. Xiang, X. Xie, H. Zhong, F. Xiao, R. Xie et al., Efficient catalytic elimination of toxic volatile organic compounds *via* advanced oxidation process wet scrubbing with bifunctional cobalt sulfide/activated carbon catalysts. Environ. Sci. Technol. **58**(20), 8846–8856 (2024). <https://doi.org/10.1021/acs.est.4c00481>
12. Z. Sun, C. Li, Z. Wei, F. Zhang, Z. Deng et al., Sulfur-bridged asymmetric CuNi bimetallic atom sites for CO_2_ reduction with high efficiency. Adv. Mater. **36**(33), e2404665 (2024). <https://doi.org/10.1002/adma.202404665>
13. K. Huang, R. Li, H. Qi, S. Yang, S. An et al., Regulating adsorption of intermediates *via* the sulfur modulating dual-atomic sites for boosting CO_2_RR. ACS Catal. **14**(11), 8889–8898 (2024). <https://doi.org/10.1021/acscatal.4c02098>
14. X. Gu, Y. Jiao, B. Wei, T. Xu, P. Zhai et al., Boron bridged NiN4B2Cx single-atom catalyst for superior electrochemical CO_2_ reduction. Mater. Today **54**, 63–71 (2022). <https://doi.org/10.1016/j.mattod.2022.02.008>
15. B. Chen, F. University, C. University, D. Shi et al., Leveraging atomic-scale synergy for selective CO_2_ electrocatalysis to CO over CuNi dual-atom catalysts. ACS Catal. **14**(21), 16224–16233 (2024). <https://doi.org/10.1021/acscatal.4c05169>
16. P. Rao, X. Han, H. Sun, F. Wang, Y. Liang et al., Precise synthesis of dual-single-atom electrocatalysts through pre-coordination-directed *in situ* confinement for CO_2_ reduction. Angew. Chem. Int. Ed. **64**(3), e202415223 (2025). <https://doi.org/10.1002/anie.202415223>
17. Y. Lin, C. Xia, Z. Zhu, J. Wang, H. Niu et al., Carbon nanocage supported asymmetrically coordinated nickle single-atom for enhanced CO_2_ electroreduction in membrane electrode assembly. Angew. Chem. Int. Ed. **64**(2), e202414569 (2025). <https://doi.org/10.1002/anie.202414569>
18. Y. Li, B. Wei, M. Zhu, J. Chen, Q. Jiang et al., Synergistic effect of atomically dispersed Ni-Zn pair sites for enhanced CO_2_ electroreduction. Adv. Mater. **33**(41), e2102212 (2021). <https://doi.org/10.1002/adma.202102212>
19. Q. Tang, Q. Hao, Q. Zhu, J. Wu, K. Huang et al., Intrinsic electron transfer in heteronuclear dual-atom sites facilitates selective electrocatalytic carbon dioxide reduction. Adv. Energy Mater. **15**(7), 2403778 (2025). <https://doi.org/10.1002/aenm.202403778>
20. Z. Zeng, L.Y. Gan, H. Bin Yang, X. Su, J. Gao et al., Orbital coupling of hetero-diatomic nickel-iron site for bifunctional electrocatalysis of CO_2_ reduction and oxygen evolution. Nat. Commun. **12**(1), 4088 (2021). <https://doi.org/10.1038/s41467-021-24052-5>
21. J. Hao, H. Zhu, Q. Zhao, J. Hao, S. Lu et al., Interatomic electron transfer promotes electroreduction CO_2_-to-CO efficiency over a CuZn diatomic site. Nano Res. **16**(7), 8863–8870 (2023). <https://doi.org/10.1007/s12274-023-5577-2>
